# Del-1, an Endogenous Inhibitor of TGF-β Activation, Attenuates Fibrosis

**DOI:** 10.3389/fimmu.2020.00068

**Published:** 2020-02-07

**Authors:** Dong-Young Kim, Seung-Hwan Lee, Yan Fu, Feifeng Jing, Won-Young Kim, Sang-Bum Hong, Jung-A Song, Han Choe, Hyun Jin Ryu, Minjung Kim, Dahae Lim, Min-Seon Kim, Chae-Ok Yun, Taewon Lee, Hoon Hyun, Eun Young Choi

**Affiliations:** ^1^Department of Biomedical Sciences, Asan Medical Center, University of Ulsan College of Medicine, Seoul, South Korea; ^2^Division of Critical Care Medicine, Department of Internal Medicine, Chung-Ang University Hospital, Seoul, South Korea; ^3^Division of Pulmonary and Critical Care Medicine, Asan Medical Center, University of Ulsan College of Medicine, Seoul, South Korea; ^4^Department of Physiology, University of Ulsan College of Medicine, Seoul, South Korea; ^5^Division of Endocrinology and Metabolism, Department of Internal Medicine, Asan Medical Center, University of Ulsan College of Medicine, Seoul, South Korea; ^6^Department of Bioengineering, College of Engineering, Hanyang University, Seoul, South Korea; ^7^Division of Applied Mathematical Sciences, College of Science and Technology, Korea University, Sejong, South Korea; ^8^Department of Biomedical Sciences, Chonnam National University Medical School, Gwangju, South Korea

**Keywords:** Del-1 (developmental endothelial locus-1), integrins, fibrosis, transforming growth factor-beta activation, inflammation

## Abstract

Uncontrolled activation of transforming growth factor (TGF)-β results in a wide range of pathologic conditions. Therapeutic interventions to regulate TGF-β signaling during fibrosis have been developed but the effectiveness is still limited. Here, we show that developmental endothelial locus-1 (Del-1) ameliorates fibrosis in mice by inhibiting α_v_ integrin-mediated activation of TGF-β. Del-1 bound to α_v_β_6_ integrin, an important activator of TGF-β, and inhibited the binding of α_v_β_6_ integrin to the latency-associated peptide (LAP), thereby suppressing α_v_ integrin-mediated activation of TGF-β. Lack of Del-1 increased colocalization of α_v_ integrin and LAP in the lungs, which was reversed by Del-1 supplementation. The crucial role of Del-1 in regulating TGF-β activity was recapitulated in a mouse model of fibrosis using an adenovirus expressing inactive TGF-β1. Del-1 supplementation improved the pathological characteristics of the mice and reduced mortality. Thus, we propose that Del-1 is a negative regulator of TGF-β activation and a potential anti-fibrotic factor.

## Introduction

Transforming growth factor (TGF-β) has a broad range of physiological and pathological effects (1). For example, it regulates cell proliferation, differentiation, and migration, and tissues- and organ-specific immune responses in a pathophysiologic state-specific manner (2). Elevated TGF-β levels are associated with progression of fibrotic and inflammatory diseases, cancers, cardiovascular diseases, diabetic nephropathy, autoimmune diseases, and neurodegenerative diseases (3–5). For this reason, TGF-β is an important therapeutic target. Several approaches have been used to inhibit TGF-β signaling in diseases related to dysregulated TGF-β signaling. First, inactive TGF-β protein mimics competitively inhibit TGF-β activation; second, molecules that interfere with the interaction between TGF-β and its receptors suppress TGF-β signaling; and third, chemical inhibition of events downstream of TGF-β receptor engagement downregulate TGF-β signaling (6, 7). However, these approaches have not been applied to patients with dysregulated TGF-β signaling due to serious side effects such as cancer development and immune dysregulation (8). This strongly suggests that to treat diseases associated with dysregulated TGF-β signaling, indirect regulation of TGF-β activation or signaling is preferable to direct blockade of TGF-β or its receptors (9–12).

TGF-β is secreted into the extracellular space as a complex called the large latent complex (LLC), which comprises latency-associated peptide (LAP) and latent TGF-β-binding protein (13). The LLC prevents TGF-β from binding to its cognate receptors (14), TGF-β receptor type-1 and−2 (TGF-βR1 and TGF-βR2). When dissociated from the LLC, TGF-β is active. TGF-β is activated by integrins, proteases, or physical stresses, such as extreme temperature, low pH, and oxidation (15). Among the numerous TGF-β activators, integrins are specific to the surrounding environment. Integrins are expressed by all nucleated cells in a cell- or tissue-specific manner; these integrins mediate different functions by binding to their respective ligands on the cell surface or extracellular matrix (ECM), or by binding to soluble glycoproteins and growth factors (16). Altered expression or activation of integrins might be involved in pathogenic processes, making this phenomenon a therapeutic target. Specifically, α_v_ integrin is normally expressed in endothelial cells and epithelial cells and is abundant in tumor cells. Additionally, its expression is increased in tumor-angiogenic blood vessels and injured epithelial cells under pathologic conditions such as fibrosis and cancer (11), although the expression levels of different α_v_ integrins vary in organ- and tissue-specific manners (17).

Pathological fibrosis is caused by abnormal regulation of ECM production in tissues or organs; uncontrolled TGF-β activation is a main component in the disease development. In particular, pulmonary fibrosis (PF) is characterized by lung stiffness due to the accumulation of excess ECM around the alveoli. This leads to shortness of breath, organ malfunction, and ultimately death (18, 19). Currently, there is no cure for PF. Of the currently available treatment options, lung transplantation is the most effective. However, because lung transplantation is limited, new and effective therapeutics are needed urgently. Overexpression of active TGF-β elicits fibrosis in animals (20), while inhibition of TGF-βR1 or R2 (or their downstream signaling mediators) suppresses fibrosis (21). Inhibiting α_v_ integrin reportedly prevents fibrosis (4, 22, 23). Integrin α_v_ binds to the arginine-glycine-aspartic acid (RGD) sequence within LAP, thereby triggering release of TGF-β from the LLC, which in turn upregulates expression of α_v_ integrin in epithelial cells to generate a self-amplifying loop that drives progression of fibrosis (4).

Developmental endothelial locus-1 (Del-1), also called Edil3, is constitutively expressed by several tissues including lung, brain, and bone, but its expression is particularly strong on endothelial cells, macrophages, and neuron. It is also secreted and associates with the cell surface or ECM. In addition, it regulates a variety of pathophysiological functions, including immune and inflammatory responses, myelopoiesis, and angiogenesis by interacting with integrins, thereby maintaining tissue homeostasis (24–28). Experiments blocking the function of a leukocyte integrin (called lymphocyte function-associated antigen 1) in models of acute and chronic inflammatory disease suggest that Del-1 acts as an inhibitor of leukocyte recruitment. Del-1 comprises three EGF domains and two discoidin domains; the second EGF domain contains an RGD sequence, a known ligand for α_v_β_3_. Previously, we showed that Del-1^−/−^ mice were more susceptible to lung injury than their wild-type (WT) littermates (29). Thus, we hypothesized that Del-1 acts as an endogenous regulator of α_v_ integrin-mediated TGF-β activation, thereby attenuating PF. This hypothesis is supported by our mechanistic findings that Del-1 prevents activation of the latent TGF-β complex by binding to α_v_β_6_ integrin and reducing collagen production. These findings have translational implications because studies in animal models of fibrosis revealed that administration of exogenous Del-1 reverses fibrosis during both the inflammatory and the fibrotic phases, thereby reducing mortality. We also show that expression of Del-1 is diminished in patients with PF. This suggests that reduced expression of Del-1 may serve as an indicator of PF development.

## Materials and Methods

### Solid-Phase Binding Assay

Binding of integrin α_v_β_6_ to immobilized Del-1 and LAP was tested in MaxiSorp 96-well plates (Nunc, Roskilde, Denmark). The plates were coated with 100 nM recombinant human Del-1 (R&D Systems), LAP (R&D Systems), or BSA in PBS at 37°C for 2 h. After washing with washing buffer (0.05% Tween-20 in PBS), the plates were blocked at room temperature (RT) for 1 h with wash buffer containing 0.5% BSA. The plates were then incubated for 2 h at 37°C in PBS containing 100 nM α_v_β_6_ (R&D Systems, catalog no. 3817-AV). To test for competitive inhibition of α_v_β_6_ integrin-LAP binding by Del-1, immobilized LAP was preincubated for 10 min at 37°C with Del-1 (100 nM), and α_v_β_6_ integrin (R&D Systems) was added (final concentration, 100 nM). After 2 h, the plates were washed three times with wash buffer and incubated at RT for 1.5 h with 100 μL of an α_v_β_6_-integrin antibody (Millipore, catalog no. MAB2077Z) (diluted 1:500 in wash buffer). After washing three times with wash buffer, 100 μL of anti-IgG-HRP (diluted 1:1,000 in wash buffer) was added and the plates were incubated at RT for 1 h. Finally, the plates were washed three times with wash buffer and incubated at RT for 0.5–1.5 h with 100 μL of tetramethylbenzidine (TMB) solution. Absorbance at 405 nm was measured in a microplate reader (Synergy HT, BioTek).

### Cell Adhesion Assay

Human small airway epithelial primary cells (PromoCell, Heidelberg, Germany) were cultured in growth medium (PromoCell, C-21070) containing SupplementMix (PromoCell, C-39175). Adhesion of HSAEpC or HEK293T cells expressing α_v_β_6_ integrin to immobilized recombinant LAP (0, 0.01, 0.1, 0.5, 1, or 10 μg/mL) or immobilized inactive TGF-β was assayed as previously described (24), with some modifications. Briefly, 96-well MaxiSorp plates were coated overnight at 4°C with 50 μL of LAP protein or with 50 μL of inactive TGF-β collected from the conditioned medium of RAW 264.7 cells expressing inactive TGF-β. The plate was blocked for 1 h with PBS containing 1% BSA and then washed with PBS. Cells that fluorescently labeled with 2′,7′-bis-(carboxyethyl)-5-(and-6)-carboxyfluorescein)-tetraacetoxymethyl ester were plated (1.5 × 10^4^ cells/well) and incubated for 20 min at 37°C in the absence or presence of Del-1 (1, 5, or 10 μg/mL), EGF_1−3_ (5 μg/mL), or an α_v_β_6_ integrin-blocking antibody (5 μg/mL). The fluorescence of the input and adherent cells was determined in a fluorescence plate reader (Synergy HT).

### Generation and Administration of Viruses Expressing Inactive TGF-β, Active TGF-β, or α_v_β_6_ Integrin

Lentiviruses and adenoviruses expressing inactive or active TGF-β were generated for use *in vitro* and *in vivo*, respectively. To prepare lentiviruses, full-length cDNA encoding a TGF-β1 precursor was obtained from C57BL/6 mouse BM-derived monocytes/macrophages stimulated with lipopolysaccharide. The WT TGF-β1 sequence was used for the inactive TGF-β gene segment, and site-directed mutagenesis of cysteine residues at 223 and 225 of the TGF-β1 preprotein to serines was performed to generate the active TGF-β gene segment (20). The sequences were verified (Macrogen, Seoul, Korea), and cloned into the *Age*I-*Xba*I sites of the pUltra lentiviral vector (Addgene, Watertown, MA, USA). Additionally, mouse *Itgav* cDNA (OriGene, Rockville, MD, USA) was cloned into the *Age*I-*Nhe*I sites of the pUltra lentiviral vector, and mouse *Itgb6* cDNA (GenScript, Piscataway, NJ, USA) was cloned into the *Age*I-*Xba*I sites. Lentivirus was generated by co-transfecting HEK 293T cells (seeded at 2 × 10^6^/100 mm dish and reaching 80–90% confluence after overnight incubation) with 5 μg of vector plasmid (pUltra), 1.25 μg of pCMV-VSVG, 2.5 μg of pMDLg/pRRE, and 1.25 μg of pRSV-Rev (Addgene) into using FuGENE HD transfection reagent (Promega, Madison, WI, USA). At 48 h post-transfection, culture supernatants were collected with a 10 mL disposable syringe and filtered through a 0.45 μm cellulose acetate filter. RAW264.7 cells (seeded in a 6-well plate at 1 × 10^6^ cells/well and incubated overnight) were infected with an 8:2 mixture of virus-containing supernatant and fresh medium supplemented with 4 μg/mL protamine sulfate. The cells were incubated for 48 h and checked for expression of green fluorescent protein (GFP) by flow cytometry or fluorescence microscopy. To prepare adenoviruses expressing inactive or active TGF-β, constructs were first cloned into the *Xho*I-*Xba*I sites of the pAdTrack-CMS vector (Stratagene, San Diego, CA, USA). Adenoviruses were generated using the AdEasy system (Stratagene) according to the manufacturer's instructions (30). Viruses were subjected to CsCl gradient purification and then dialyzed in Dulbecco's phosphate-buffered saline. The viral titre was adjusted to 10^9^ plaque-forming units/mL, and 40 μL of the virus suspension was administered intratracheally to each mouse. The infection efficiency was verified by infecting mouse embryonic fibroblasts isolated from WT and Del-1^−/−^ mice at a multiplicity of infection of 10 and then evaluating the infected cells for GFP expression by flow cytometry after 24 h. More than 80% of cells used to generate viruses were GFP-positive.

### *In vitro* Measurement of α_v_ Integrin-Mediated TGF-β Activation

MaxiSorp 96-well plates were coated with α_v_β_3_ integrin (5 μg/mL in PBS containing 1 mM MnCl_2_) at 37°C for 3 h. Mn^2+^ was used to induce active integrins (31, 32). After aspirating the residual liquid, RAW264.7 cells (2 × 10^4^ cells/well) expressing inactive TGF-β were plated in 100 μL of DMEM-10 and incubated in a CO_2_ incubator at 37°C for 16 h. The cells were then treated with 1 mM MnCl_2_ for 4 h at 37°C. Some wells were pretreated for 2 h with recombinant human Del-1 (5 μg/mL, R&D Systems) or mouse EGF_1−3_ (5 μg/mL) prior to the addition of MnCl_2_. Active TGF-β concentrations were measured by ELISA (eBioscience). The supernatant collected from RAW264.7 cells lentivirally expressing active TGF-β was used as a positive control. In other experiments, HEK293T cells expressing α_v_β_6_ integrin, which was verified by flow cytometry using anti-αvβ6 antibody (clone 10D5; Millipore), were plated into 24-well plates (Nunc) at 1 × 10^5^ cells/well and incubated at 37°C overnight. The cells were treated with 200 μL of inactive TGF-β collected from the conditioned medium of RAW264.7 cells lentivirally expressing inactive TGF-β and then incubated in a CO_2_ incubator at 37°C for 16 h. Some cells were co-treated with recombinant human Del-1 (0, 1, 5, 10, or 20 μg/mL), mouse EGF_1−3_ (10 μg/mL), or an α_v_-blocking antibody (2.2 μg/mL; clone AV1; Millipore). Active-TGF-β concentrations were measured by ELISA. To measure α_v_β_6_ integrin-mediated TGF-β activation in a cell-to-cell contact *in vitro* system, HEK293T cells expressing α_v_β_6_ integrin and HEK293T cells expressing inactive TGF-β were co-cultured at a 1:1 ratio (each cell line plated at 2 × 10^4^ cells/well) in the presence of recombinant human Del-1 (0, 1, 5, and 10 μg/mL) or an anti-α_v_β_6_-blocking antibody (5 μg/mL; clone 10D5; Millipore) at 37°C overnight. The supernatants were analyzed for active TGF-β by ELISA. 200 μL of inactive TGF-β collected from the conditioned medium of RAW264.7 cells lentivirally expressing inactive TGF-β was used to coat a MaxiSorp 96 well plate at 4°C overnight. After removing the liquid, HEK293T cells expressing α_v_β_6_ integrin or HSAEpC (C-12642; PromoCell) were plated (2.5 × 10^4^ cells/well) and incubated in the presence of recombinant human Del-1 (1, 5, and 10 μg/mL), EGF_1−3_ (5 μg/mL), or anti-α_v_β_6_-blocking antibody (5 μg/mL) at 37°C for 16 h. The concentration of active TGF-β was measured by ELISA.

### ELISA to Detect Active TGF-β and LAP

Activation of TGF-β in cell culture experiments or in BALF obtained from WT and Del-1^−/−^ mice with PF induced by inactive TGF-β-expressing adenovirus was measured using the human/mouse TGF-β1 ELISA Ready-SET Go kit (eBioscience) according to the manufacturer's protocol. Briefly, the plate was coated overnight at 4°C with capture antibody, washed five times with PBST (0.05% Tween 20 in PBS), blocked with 1× assay buffer, incubated at RT for 1 h, and washed five times with 0.05% PBST. The plate was then loaded with samples and standards, incubated at RT for 2 h or at 4°C overnight, washed five times with 0.05% PBST, and incubated at RT for 1 h with biotin-conjugated anti-active TGF-β antibody. After washing five times with 0.05% PBST, the plate was incubated at RT for 30 min with avidin-conjugated HRP. The plate was washed five times with 0.05% PBST, developed with TMB, and read at 630 nm on a microplate reader (Synergy HT). The concentrations of LAP were measured as described above but using a mouse LAP (TGF beta 1) Ready-SET Go kit (eBioscience).

### Bleomycin-Induced Fibrosis

C57BL/6 mice (C57BL/6NCrljOri) were purchased from Orient Bio (Seongnam, Gyeonggi Province, Korea). Del-1^−/−^ mice on a C57BL/6 background were kindly provided by Prof. T. Chavakis (Dresden University, Germany) (24). Sex- and age-matched mice were used for the experiments. Six- to eight-week-old WT and Del-1^−/−^ mice were anesthetised by intraperitoneal injection of avertin solution (0.5 mL, containing tribromethanol and amylalcohol) (Sigma-Aldrich, St. Louis, MO, USA). Forty microliters of BLM (Sigma-Aldrich, B5507; MB Cell, MB-B4252) were injected into the trachea. Different concentrations (0.7–2 U/kg) of BLM were used for some experiments because BLM activity differed according to the provider and the lot number. At the indicated dpa, the BALF and lung tissues were collected for analysis. To induce systemic fibrosis in WT and Del-1^−/−^ mice, 100 μL of BLM (8 U/kg/dose; Sigma-Aldrich, B5507) were injected subcutaneously on a daily basis for 15 days, as previously described (33). Skin and lung tissues were collected at 23 dpa after the first BLM injection.

### Supplementation With Del-1 Protein

WT and Del-1^−/−^ mice with BLM-induced PF were supplemented with soluble mouse Del-1 (sDel-1; EGF_1−3_) or human Del-1-Fc at multiple time-points following BLM instillation. In some experiments, bovine serum albumin (BSA; Sigma-Aldrich) or mouse Del-1 was administered intravenously at 5, 9, and 13 dpa (50 μg/dose/mouse). In other experiments, Fc (Y-Biologics, Daejeon, Korea) or human Del-1-Fc protein was administered at 5, 9, and 13 dpa; at 9 and 13 dpa; or at 14 and 18 dpa (each at 50 μg/dose/mouse). The mice were monitored for survival and sacrificed at 21 dpa for collection of lung tissues, *ex-vivo* μ-CT, immunohistochemistry, and collagen production assays. Human Del-1-Fc fusion protein (Del-1-Fc) purified from HEK 293F cells was purchased (DEL-1-C-Fc; Y-Biologics). To generate EGF_1−3_, a fragment of the three EGF domains (amino acids 17–157) of the mouse Del-1 gene (clone BC056386; Open Biosystems, Lafayette, CO, USA) was cloned into the pENTR1A vector (Thermo Fisher Scientific), transferred into a vector containing a maltose-binding protein (MBP) tag (pDEST-MBP), and expressed in *Escherichia coli* BL21. Cultivated-cell lysate was purified by immobilized-metal ion affinity chromatography (IMAC), filtered through a 0.45-μm Whatman syringe filter (GE Healthcare, Little Chalfont, UK), and loaded onto a HisTrap HP column (GE Healthcare). After elution, the protein was treated with tobacco etch virus protease and 1 mM dithiothreitol to cleave the MBP tag, purified a second time by IMAC, and passed through a HisTrap HP column. To improve the purity of the protein, the protein solution was concentrated using a Centricon filter (Millipore, MA, USA) and separated by Superdex-75 gel filtration chromatography. The concentrated protein was loaded onto an XK 26/100 column (GE Healthcare) packed with Superdex-75 HR (GE Healthcare). All purified fractions were analyzed by sodium dodecyl sulfate-polyacrylamide gel electrophoresis (SDS-PAGE) on 10% Tris-Tricine gels. The purified mouse Del-1 protein was subjected to matrix-assisted laser desorption/ionization-time of flight (MALDI-TOF) and MALDI-TOF-TOF mass spectrometry on a Voyager-DE STR mass spectrometer (Applied Biosystems, Foster City, CA, USA) and a 4700 MALDI-TOF/TOF analyzer (AB Sciex, Framingham, MA, USA), respectively. Data were analyzed with Data Explorer software (Applied Biosystems). The peptide masses obtained were searched against the NCBI database using the Mascot peptide mass fingerprinting search program (Matrix Science, Boston, MA, USA). The final protein was tested with the Silver Stain Plus kit (Bio-Rad Laboratories, Hercules, CA, USA). Endotoxins were removed with 1% Triton X-114 (Sigma-Aldrich), and the residual endotoxin level was measured in a quantitative Endpoint Chromogenic Limulus Amebocyte Lysate assay (Lonza, Basel, Switzerland) to ensure that the purified mouse Del-1 was endotoxin-free.

### *Ex vivo* Micro-Computed Tomography (μ-CT) Analysis

Mice were sacrificed, and the lungs insufflated and fixed with 4% paraformaldehyde, incubated with a 1:1 solution of glutaraldehyde:acetone (Sigma-Aldrich) for 12 h, dehydrated in 99.9% ethanol for 12 h, and transferred to 99% hexamethyldisilazane (Sigma-Aldrich) for air drying. Inverted lungs were then placed in a container fashioned from the bottom tip of a 15-mL conical tube and covered with Parafilm. The tip was then secured to the plate of the scanner. Lungs were scanned using a SkyScan 1172 (Kontich, Belgium), which is a high-resolution X-ray μ-CT system. The scan settings were as follows: 40 kV, no filter, X-ray beam, and at 2,000 × 1,000 pixels (8.4 μm/pixel) for camera binning. Images were collected at four frames per every 0.2° angular rotation, with an exposure time of 295 ms. All frames were reconstructed using NRecon software. Representative μ-CT sections from each mouse were extracted for analysis. To quantify fibrosis in the lung sections, areas showing highlighted grayscale densities (according to a manually selected grayscale threshold value that best distinguished the fibrotic areas from the background image) were highlighted in red, and the total fibrotic area was calculated by dividing the red area by the total area of the lung section image and multiplying by 100. The calculations were performed using Image J software (NIH, Bethesda, MD, USA).

### Measurement of Blood Oxygen Levels

Mice were anesthetised by intraperitoneal injection of 0.5 mL avertin solution and blood was collected from the heart into a heparinized 1 mL syringe. The blood was loaded onto an automatic blood gas and electrolyte analyzer (GEM Premier 3000, Instrumentation Laboratory, Bedford, MA, USA) to measure oxygen levels.

### Immunohistochemistry

To analyze colocalization of α_v_ and LAP in the lungs, WT and Del-1^−/−^ mice with BLM-induced PF were administered Fc or Del-1-Fc intravenously at 5, 9, and 13 dpa. At day 21 dpa, the mice were transcardially perfused with PBS, the lungs fixed with 4% paraformaldehyde and embedded in OCT compound, and 15 μm cryostat sections prepared. The frozen sections were air-dried, washed three times with PBS (5 min per wash), permeabilized for 10 min with 0.1% Triton X-100 in PBS, and then blocked for 2 h with PBS containing 2% normal goat serum (Thermo Fisher Scientific) and 10% BSA. After washing three times (10 min per wash), primary antibodies specific for α_v_ (diluted 1:500; Millipore) and LAP (diluted 1:500; Santa Cruz Biotechnology, Dallas, TX, USA) were added and sections were incubated at 4°C for 12 h, followed by a further 1 h incubation with the cognate secondary antibodies. After washing three times (each for 10 min), Fluoromount G (Electron Microscopy Sciences, Hatfield, PA, USA) was added and the slides were analyzed by confocal microscopy (LSM 710; Zeiss, Oberkochen, Germany). To confirm the proximity of α_v_ integrins and LAP on epithelial cells and fibroblasts in the lung sections, immunohistochemistry was performed as described above using primary antibodies specific for α_v_ integrin (Millipore Sigma), LAP (Santa Cruz Biotechnology), E-cadherin (Cell Signaling, Danvers, MA, USA), and/or α-SMA (Abcam), followed by cognate secondary antibodies.

### Measurement of Collagen Production

Lung collagen content was quantified by measuring lung hydroxyproline levels with a hydroxyproline assay kit (Sigma-Aldrich) according to the manufacturer's instructions. Briefly, 10 mg of lung lysate was transferred to a microcentrifuge tube containing 100 μL of water, treated with 100 μL of 12 M hydrochloric acid, and hydrolysed at 120°C for 3 h. The supernatant (10 μL) was transferred to a 96-well plate and evaporated in an oven at 60°C. The wells were incubated at RT for 5 min with 100 μL of Chloramine T/Oxidation Buffer mixture, and then incubated at 60°C for 90 min with 100 μL of diluted 4-(dimethylamino) benzaldehyde. The absorbance was measured at 560 nm. The hydroxyproline content in the lungs was determined based on total lung weight.

### Western Blot Analysis

Samples were prepared in radioimmunoprecipitation assay (RIPA) buffer, boiled, and separated by SDS-PAGE on 10% polyacrylamide gels, followed by transfer to Immobilon-P membranes (Millipore) using the Trans-Blot semi-dry electrophoretic transfer system (Bio-Rad). Membranes were blocked for 1 h at RT with 5% skim milk in TBS containing 0.1% Tween 20 (TBST) and then incubated at 4°C overnight with primary antibodies specific for α-SMA (Sigma-Aldrich), tubulin (Cell Signaling), phospho-Smad2 (Cell Signaling), phospho-Smad3 (Abcam), Smad2 (Cell Signaling), Smad3 (Abcam), integrin beta 3 (Abcam), integrin beta 5 (Abcam), integrin beta 6 (R&D systems), and β-actin (Cell Signaling) (all diluted in 2% skim milk in TBST). Membranes were washed and then incubated with HRP-conjugated goat anti-mouse, goat anti-rabbit, or donkey anti-goat IgG secondary antibodies (Jackson ImmunoResearch, Suffolk, UK) diluted in 2% skim milk in TBST. Blots were developed with the SuperSignal West Pico chemiluminescent substrate system (Thermo Fisher Scientific).

### Immunoprecipitation

HEK 293T cells expressing α_v_β_6_ integrin (induced lentiviral expression as described above) were cultured in 100 mm dishes and incubated for 6 h at 37°C with Del-1 (1 μg/mL; R&D Systems) and/or LAP (0.5 μg/mL, R&D Systems). A previously described protocol was used (34), with some modifications. Briefly, the cells were washed with ice-cold PBS and lysed for 20 min in 900 μL of buffer containing 1% 3-[(3-cholamidopropyl)dimethylammonio]-1-propanesulfonate (CHAPS), 30 mM Tris-HCl, pH 7.5, 150 mM NaCl, and complete protease inhibitor cocktail, Next, the mixture was centrifuged at 19,000 × g for 15 min. Cell lysates were precleared for 2 h at 4°C with Protein G Mag Sepharose (GE Healthcare), incubated overnight at 4°C with an anti-Flag antibody (diluted 1:200; OriGene), and then incubated for 5 h with 25 μL of Protein G Mag Sepharose. Complexes containing antibody-bound Ag and coprecipitated proteins were pelleted and washed twice with 1% CHAPS buffer, and then three times with TBS. Bound proteins were eluted with 50 μL of 1× RIPA sample buffer and analyzed by western blotting with antibodies specific for Del-1 (Proteintech), LAP (Santa Cruz Biotechnology), Flag (OriGene), and β-actin (Cell Signaling Technology).

### Conventional and Quantitative Reverse-Transcription (RT)-PCR

Conventional and real-time RT-PCR was performed using cultured primary fibroblasts isolated from WT and Del-1^−/−^ mice. Total RNA was isolated using QIAzol (Qiagen, Hilden, Germany), and cDNA was synthesized using the High Capacity cDNA Reverse Transcription Kit (Applied Biosystems/ThermoFisher Scientific). The cDNA was amplified using LightCycler 480 SYBR Green 1 Master Mix and a LightCycler 480 machine (Roche, Mannheim, Germany). The following PCR conditions were used: 95°C for 15 min; 50 cycles of 30 s at 95°C, 30 s at 60°C, and 30 s at 72°C; and 95°C for 15 min. Melting curve analysis was conducted for all PCR products to ensure primer specificity. Del-1 mRNA levels were normalized to those of 18S mRNA, and relative transcript levels were determined using the comparative C_T_ method (35). For conventional RT-PCR, the Quick Taq HS DyeMix (Toyobo, Osaka, Japan) was used with the following PCR conditions: 94°C for 2 min, followed by 30–32 cycles of 30 s at 94°C, 30 s at 60°C, and 30 s at 68°C; with a final extension at 68°C for 3 min. The primer sequences were as follows: mDel-1 forward primer: 5′-CCT GTG AGA TAA GCG AAG-3′; mDel-1 reverse primer: 5′-GAG CTC GGT GAG TAG ATG-3′; mouse α_v_ integrin forward primer: 5′-CAC TTT GGG CTG TGG AAT CG-3′; mouse α_v_ integrin reverse primer: 5′-TGC CAA GAT GAT CAC CCA CA-3′; mIL-1β forward primer: 5′-CTT TCC CGT GGA CCT TCC AG-3′; mIL-1β reverse primer: 5′-ATA TGG GTC CGA CAG CAC GA-3′; mTNF-α forward primer: 5′-CCA AAT GGC CTC CCT CTC AT-3′; mTNF-α reverse primer: 5′-TCC AGC TGC TCC TCC ACT TG-3′; mIL-6 forward primer: 5′-TGG GAC TGA TGC TGG TGA CA-3′; mIL-6 reverse primer: 5′-GCC TCC GAC TTG TGA AGT GGT-3′; mCol1a forward primer: 5′-ACG ATG GTG CTG TTG GTG CT-3′; mCol1a reverse primer: 5′-CCT TTA GCG CCA GGT TGT CC-3′; mβ3 integrin forward primer: 5′-GGG ATG ACA TCG AGC AGG TG-3′; mβ3 integrin reverse primer: 5′-CAA GGC CAA TGA GCA GGA TG-3′; mβ6 integrin forward primer: 5′-TGT GAC TGC GAC TGC CAG AG-3′; mβ3 integrin reverse primer: 5′-CTC GCA GTG AGG ACC CAT GT-3′; mGAPDH forward primer: 5′-GCC ACC CAG AAG ACT GTG GAT-3′; mGAPDH reverse primer: 5′-GGG ATG ACC TTG CCC ACA G-3′; m18S forward primer: 5′-CGC GGT TCT ATT TTG TTG GT-3′; and m18S reverse primer: 5′-AGT CGG CAT CGT TTA TGG TC-3′.

### Isolation and Culture of Primary Lung Fibroblasts

Primary lung fibroblasts were isolated as described previously (36) with some modifications. Briefly, lungs from WT and Del-1^−/−^ mice were minced, treated for 1 h at 37°C with type I collagenase (0.5 mg/mL, Gibco, Grand Island, NY, USA), and passed through a 100 μm cell strainer. Cells were cultured in Dulbecco's modified Eagle's medium (DMEM)-10 until confluent. Lung fibroblasts were used at passage two.

### *In vitro* Proliferation and Differentiation Assays

To assess cell proliferation *in vitro*, primary lung fibroblasts (2 × 10^5^ cells/well) were plated in a 6-well plate containing DMEM-10. After removing the culture medium, the cells were loaded for 20 min at 37°C with CellTrace carboxyfluorescein succinimidyl ester (CFSE; ThermoFisher Scientific) diluted in pre-warmed PBS (2.5 μM). The cells were washed twice with culture medium to remove excess CFSE and then resuspended in fresh, pre-warmed DMEM-10. The cells were then treated for 48 h with mouse TGF-β1 (5 ng/ml; R&D Systems). The cells were detached using 0.05% trypsin/EDTA and analyzed by flow cytometry. To evaluate differentiation of lung fibroblasts, primary cells were plated overnight at 37°C to allow attachment as a monolayer. Following 24 h of incubation in culture medium containing 2.5% serum, cells were treated with mouse TGF-β1 (5 ng/ml) or vehicle. After 48 h, cells were rinsed with DPBS and detached using 0.05% trypsin/EDTA. The cells were fixed for 30 min at RT with 2% PFA in PBS, permeabilized with 0.05% Triton X-100 in FACS buffer (PBS containing 0.1% BSA and 0.1% sodium azide), and analyzed for expression of α-SMA by flow cytometry.

### Measurement of Apoptosis

The BAL fluid cells were stained using an annexin V apoptosis detection kit (BD Pharmingen). The cells were then incubated with antibodies specific for Gr-1 (Biolegend) and E-cadherin (Biolegend), followed by flow cytometry.

### Measurement of Del-1 Concentrations

The concentration of Del-1 in human plasma from healthy controls and patients with PF was measured in an ELISA. MaxiSorp 96-well plates (Nunc, Roskilde, Denmark) were coated with 50 μL of l-α-phosphatidylserine (200 ng/mL; Avanti Polar Lipids, Inc., Alabaster, AL, USA) at 4°C for 12 h (37). After washing three times with 0.05% PBS-Tween-20 (PBST), 100 μL of plasma (diluted 1:5 in PBS) was added, and the plate was incubated at room temperature (RT) for 4 h. Serially diluted recombinant human Del-1 protein (R&D Systems, Minneapolis, MN, USA) was used as the standard. The plate was then washed, incubated at RT for 2 h with a rabbit anti-Del-1 antibody (diluted 1:500; Proteintech, 12580-1-AP, Rosemont, IL, USA), washed four times with PBST, and incubated at RT for 1 h with horseradish peroxidase (HRP)-conjugated anti-rabbit IgG (1:1,000 dilution; Jackson ImmunoResearch, West Grove, PA, USA). After five washes with PBST, the plate was incubated with tetramethylbenzidine substrate (BD Biosciences, San Diego, CA, USA). Absorbance at 650 nm was read on a microplate reader (Synergy HT, BioTek, Winooski, VT, USA). Alternatively, human plasma was added to MaxiSorp 96-well plates and incubated overnight at 4°C. After two washes with 0.05% PBST, the plate was incubated at RT for 2 h with rabbit anti-Del-1 antibody (Proteintech; 12580-1-AP), and developed as described above. For other experiments, BALF and serum were collected from WT mice with BLM-induced PF on the indicated days. Total protein content in the BALF was determined by a bicinchoninic acid (BCA) colorimetric assay (Thermo Scientific, Rockford, IL). Mouse Del-1 levels were measured in 100 μL of BALF or serum (diluted 1:5 in PBS) as described for measurement of human Del-1.

### Study Approval

Animal studies were approved by the ASAN Institute for Life Sciences Institutional Animal Care and Use Committee (Project number: 2016-12-039). Human plasma from healthy control and PF patients was provided by the Collaborative Biobank of Korea at Soonchunhyang University Bucheon Hospital, Korea. All materials and clinical information used for research purposes (without personal identification) were collected from donors by the biobank; all donors provided written informed consent. Human studies were approved by the ASAN Medical Center Institutional Review Board (IRB no: 2017-1340) and Soonchunhyang University Bucheon Hospital Institutional Review Board (IRB no: schbc-biobank-2017-013-01).

### Statistical Analysis

Data were compared using a two-tailed Student's *t*-test, or non-parametric statistical analysis (Mann-Whitney *U*-test) when datasets from two comparison groups showed a non-normal distribution in the Kolmogorov-Smirnov test. For some experiments, a small number of samples (*n* = 3) was used due to limited availability (the PF mice had often died at the time points analyzed), in which asterisks indicate statistical significance when all values in the treatment group were larger or smaller than those for the control group. In such cases, the *p*-value = 0.049535 (Mann-Whitney *U*-test). Other statistical analyses methods used were: (i) linear regression analysis to evaluate dose-dependent inhibition of TGF-β activation by Del-1 (**Figure 2B**); (ii) log-rank test to compare survival of the groups (**Figure 5B**); and (iii) Dunnett's test to compare the healthy control group with the IPF patient group or to compare different IPF patient groups (**Figure 6A**).

## Results

### Del-1 Interferes With Binding of α_v_ Integrin to LAP

**Figure 1:**
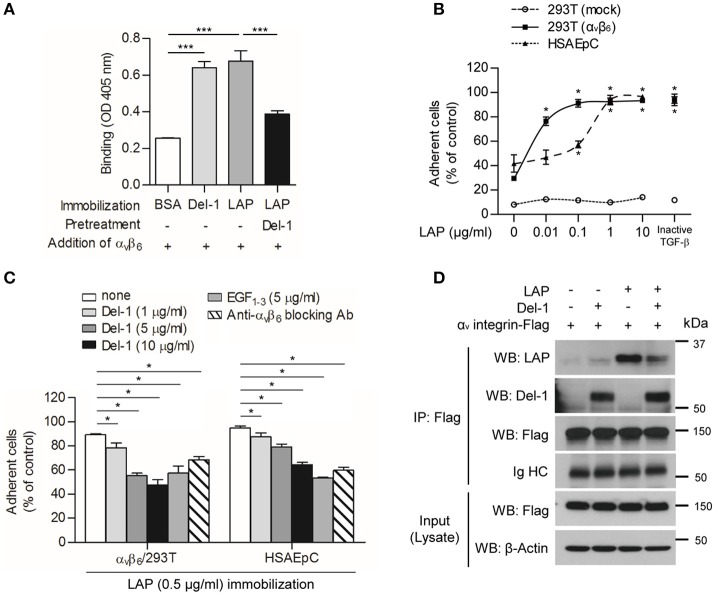
Del-1 interferes with binding of α_v_ integrin to LAP. **(A)** Binding of α_v_β_6_ integrin (100 nM) to immobilized BSA, Del-1 (100 nM), and LAP (100 nM), and binding of α_v_β_6_ integrin to immobilized LAP in the presence of Del-1, as assessed in a solid-phase binding assay. Data are representative of three independent experiments, each with similar results, and are expressed as the mean ± SD (*n* = 4 per group). ***p < 0.001; Student's *t*-test. **(B)** Adhesion of α_v_β_6_-overexpressing HEK293T cells or human small airway alveolar epithelial primary cells (HSAEpCs) onto increasing concentrations of immobilized LAP protein was assessed. Alternatively, adhesion of cells to immobilized inactive TGF-β derived from conditioned medium was assessed. Data are expressed as the mean ± SD (*n* = 3). *p < 0.05; Mann-Whitney *U*-test. **(C)** Adhesion of α_v_β_6_ integrin-overexpressing HEK 293T cells or HSAEpC to immobilized LAP is impaired by Del-1 (1, 5, and 10 μg/mL), EGF_1−3_ (5 μg/mL), or an α_v_β_6_-blocking antibody (5 μg/mL). Data are expressed as the mean ± SD (*n* = 3). *p < 0.05; Mann-Whitney *U*-test. **(D)** Representative co-immunoprecipitation assay to validate the interaction between α_v_ integrin and Del-1 and the interaction between α_v_ integrin and LAP in the absence or presence of Del-1. HEK293T cells overexpressing α_v_β_6_ integrin after lentiviral transduction were incubated for 6 h with Del-1 (20 nM) or LAP (20 nM) and then processed for immunoprecipitation and western blotting. Data are representative of three independent experiments, each with similar results.

To determine whether Del-1 is involved in TGF-β activation, we first studied the effect of Del-1 on binding of α_v_ integrins α_v_β_3_ and α_v_β_6_ to LAP; the latter is expressed exclusively by lung epithelial cells, to LAP. We found that integrin α_v_β_3_ bound to immobilized Del-1 and LAP, and that pretreatment with Del-1 led to a reduction in binding between α_v_β_3_ integrin and LAP (Figure S1A). Likewise, Del-1 bound to both immobilized α_v_β_3_ integrin and LAP, and pretreatment with α_v_β_3_ integrin inhibited binding between Del-1 and LAP (Figure S1B). Interestingly, integrin α_v_β_6_ bound to immobilized Del-1 and LAP (Figure 1A). Pretreatment with Del-1 reduced binding between α_v_β_6_ integrin and LAP significantly (Figure 1A). α_v_β_6_-overexpressing HEK293T cells and human small airway alveolar epithelial primary cells (HSAEpC) became more adherent as the concentration of recombinant LAP increased (Figure S2 and Figure 1B), while Del-1 inhibited adhesion in a dose-dependent manner and as effectively as a α_v_β_6_-blocking antibody (Figure 1C). A mutant form of Del-1 that lacks two discoidin domains (EGF_1−−3_; an amino-terminal fragment containing the amino acids 17–157) showed results similar to those of WT Del-1 (Figure 1C). To confirm these findings, we performed an immunoprecipitation assay using HEK293T cells expressing α_v_β_6_ integrin. We found that α_v_ integrin bound to Del-1 and LAP (Figure 1D); thus, we conclude that Del-1 inhibits binding between α_v_ integrin and LAP.

### Del-1 Inhibits α_v_ Integrin-Mediated Activation of TGF-β *in vitro*

**Figure 2:**
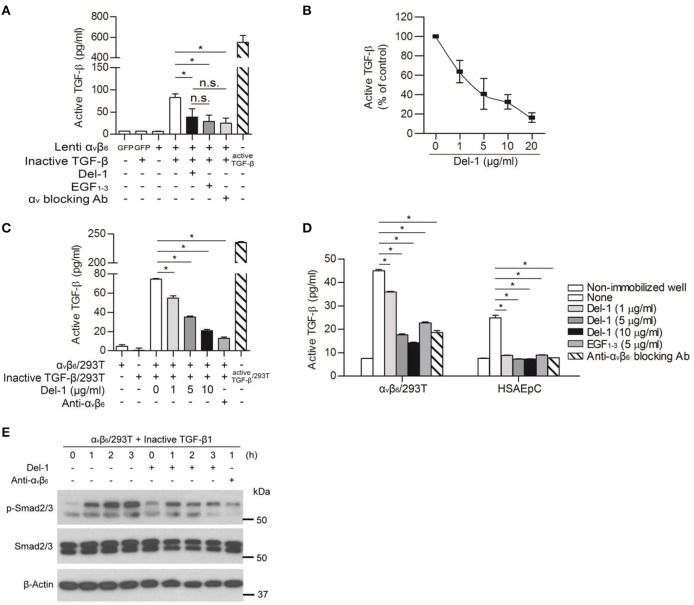
Del-1 inhibits α_v_ integrin-mediated activation of TGF-β *in vitro*. **(A)** Activation of inactive TGF-β by α_v_β_6_ integrin lentivirally expressed by HEK293T cells in the absence or presence of Del-1 (100 nM) or an α_v_-blocking antibody (2.2 μg/mL). Integrin α_v_β_6_-overexpressing HEK293T cells (1 × 10^5^/well) were plated and incubated overnight at 37°C. Conditioned medium from RAW264.7 cells overexpressing inactive TGF-β was then added in the presence of Del-1 or an α_v_-blocking antibody. EGF_1−3_ (10 μg/mL) was added to some wells instead of recombinant Del-1. After 12 h, active TGF-β levels were measured in an ELISA. Active TGF-β-containing supernatant was used as a positive control. Data are representative of three independent experiments, each with similar results, and are expressed as the mean ± SD (*n* = 3 per group). *p < 0.05; n.s., not significant; Mann-Whitney *U*-test. **(B)** Relative inhibition of α_v_β_6_ integrin-mediated TGF-β activation by increasing concentrations of Del-1. α_v_β_6_ integrin-overexpressing HEK293T cells (1 × 10^5^/well) were plated and incubated overnight at 37°C. Conditioned medium from RAW264.7 cells overexpressing inactive TGF-β was added in the presence of Del-1 (0, 1, 5, 10, or 20 μg/mL). After 12 h of incubation, active TGF-β levels were measured in an ELISA. Data are presented as a percentage relative to the concentration of active TGF-β in the absence of Del-1, which was set to 100%. Data are representative of three independent experiments, each with similar results, and are expressed as the mean ± SD (*n* = 3 per group). Linear regression analysis revealed a significant inverse correlation between TGF-β activation and Del-1 concentration (*p* = 0.0002, *R*^2^ = 0.6301). **(C)** Inhibition of TGF-β activation in co-cultures of cells expressing either α_v_β_6_ integrin or inactive TGF-β by increasing concentrations of Del-1. α_v_β_6_ integrin-overexpressing HEK293T cells (2 × 10^4^/well) and inactive TGF-β-expressing HEK293T cells (2 × 10^4^/well) were plated and then incubated overnight at 37°C in the presence of Del-1 (0, 1, 5, or 10 μg/mL) or an α_v_β_6_-blocking antibody (5 μg/mL). The supernatants were analyzed in an ELISA to measure active TGF-β. Data are expressed as the mean ± SD (*n* = 3 per group). *p < 0.05; Mann-Whitney *U*-test. **(D)** Inhibition of TGF-β activation by Del-1 in α_v_β_6_ integrin-overexpressing HEK293T cells or primary epithelial cells exposed to immobilized inactive TGF-β. Conditioned medium from RAW264.7 cells overexpressing inactive TGF-β were coated onto the plate, and then α_v_β_6_ integrin-overexpressing HEK293T cells or HSAEpCs were plated in the presence of Del-1, EGF_1−3_, or an α_v_β_6_-blocking antibody. After an overnight incubation at 37°C, active TGF-β levels were measured in an ELISA. Data are representative of three independent experiments, each with similar results, and are expressed as the mean ± SD (*n* = 3 per group). *p < 0.05; Mann-Whitney *U*-test. **(E)** Del-1-mediated reduction in the amount of active TGF-β to weaken TGF-β signaling in cells. Representative western blots showing phospho-Smad2/3 in HEK 293T cells overexpressing α_v_β_6_ integrin in response to inactive TGF-β. Cells were incubated at 37°C with a RAW 264.7 cell-derived conditioned medium containing inactive TGF-β in the presence of Del-1 for 1–3 h, or in the presence of an α_v_β_6_-blocking antibody for 1 h. Data are derived from three independent experiments.

Next, we checked whether the inhibitory effect of Del-1 on the interaction between α_v_ integrin and LAP controls activation of TGF-β. To induce integrin-mediated conversion of TGF-β from an inactive to an active form *in vitro*, we generated supernatants containing inactive (WT) TGF-β or active (mutant) TGF-β. Both inactive and active TGF-β-containing supernatants contained LAP; of note, very little active TGF-β was detected in the inactive TGF-β-containing supernatants (Figure S3). To ascertain whether Del-1 inhibits α_v_ integrin-mediated TGF-β activation *in vitro*, HEK293T cells expressing α_v_ integrin were incubated with immobilized inactive TGF-β and the concentrations of active TGF-β were measured in an enzyme-linked immunosorbent assay (ELISA). Incubation of cells expressing α_v_β_6_ integrin with inactive TGF-β resulted in TGF-β activation; this was suppressed by adding recombinant Del-1 (the suppressive effect was comparable to that observed with a α_v_-blocking antibody) (Figure 2A). Inhibition of α_v_β_6_ integrin-mediated activation of TGF-β by Del-1 was concentration-dependent (Figure 2B). The results for EGF_1−3_ were similar to those for WT Del-1, suggesting that the EGF domains are sufficient to regulate TGF-β activation (Figures 2A,B). Likewise, incubation of RAW264.7 mouse macrophages overexpressing inactive TGF-β, which is primarily produced by infiltrating macrophages during PF, with immobilized α_v_β_3_ integrin resulted in TGF-β activation; this was suppressed by addition of recombinant Del-1 (Figure S4).

Because cell-to-cell contact is important for TGF-β activation during fibrosis (15), we used inactive TGF-β-overexpressing HEK293T cells instead of the free form of latent TGF- β and repeated the experiment shown in Figure 2A. Analogous to the data obtained using the free form of latent TGF-β in the well (Figures 2A,B), we found that Del-1 significantly inhibited the α_v_β_6_-mediated activation of TGF-β significantly in co-cultures of inactive TGF-β-overexpressing HEK293T cells and α_v_β_6_-overespressing HEK293T cells; this effect was dose-dependent (Figure 2C). Furthermore, Del-1 decreased the conversion of inactive immobilized TGF-β to active TGF-β in α_v_β_6_-overexpressing HEK293T cells or HSAEpC, as did the α_v_β_6_-blocking antibody (Figure 2D). This Del-1-mediated reduction in the amount of active TGF-β weakened Smad2/3 phosphorylation in α_v_β_6_-expressing HEK293T cells (Figure 2E). Taken together, these findings suggested that Del-1 competes with LAP for binding to α_v_-integrin, thereby inhibiting activation of TGF-β *in vitro*.

### Del-1 Regulates Interaction Between α_v_ Integrin and LAP *in vivo*

Bleomycin (BLM)-induced PF is a simple and commonly used-animal model of PF, in which an inflammatory phase is followed by a fibrotic phase. Although there is some variability in this model depending on the BLM concentrations used, inflammatory cell migration can be detected on Day 3, which then peaks on Day 7; collagen deposition increases gradually, beginning on Day 14 and peaking on Day 21 (38–40).

The model was established as shown in Figure S5. In the mouse BLM-induced PF model, the amount of Del-1 mRNA in lung tissue fell at the final stage of fibrosis, although there was a transient increase during the early phase (Figure S5A). The levels of Del-1 in the serum and bronchoalveolar lavage fluid (BALF) decreased significantly as the disease progressed. The reduction in Del-1 expression correlated inversely with the level of collagen production at the final stage of fibrosis (Figure S5B). The levels of active TGF-β, α_v_ integrin, and inflammatory mediators were altered during the course of BLM-induced PF (Figures S5C–E).

**Figure 3:**
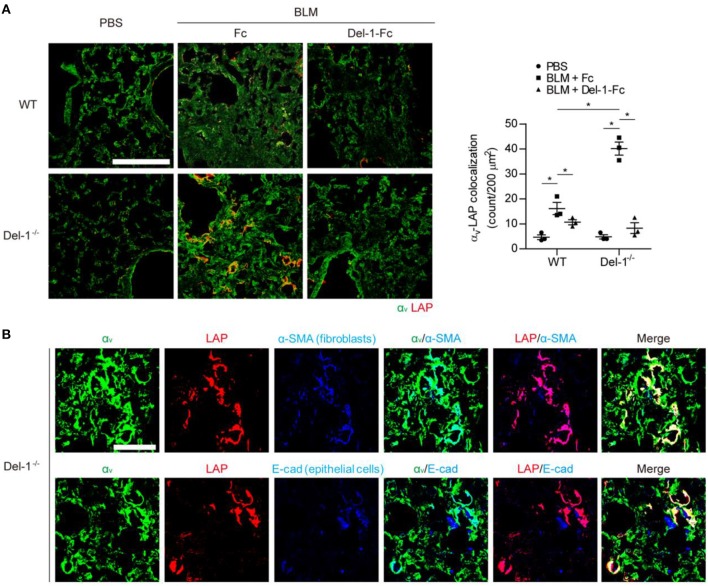
Endogenous and exogenous Del-1 inhibit the interaction between α_v_ integrin and LAP *in vivo*. **(A)** Representative lung sections showing colocalization of α_v_ integrin with LAP in WT and Del-1^−/−^ mice with BLM-induced PF supplemented with control Fc or Del-1-Fc (intravenous injection; 50 μg/dose Fc or Del-1-Fc on Days 5, 9, and 13 post-BLM instillation; *n* = 3 mice/group). Lung samples were collected at 21 dpa. Green, α_v_ integrin; red, LAP. Scale bar = 200 μm. Quantification of the colocalization is shown in the right panel. In each section, the number of double-positive cells per 200 μm^2^ was counted. Data are expressed as the mean ± SEM. *p < 0.05; Mann-Whitney *U*-test. **(B)** Representative images showing colocalization of α_v_ integrin with LAP in fibroblasts or epithelial cells in lungs from Del-1^−/−^ mice with BLM-induced PF (*n* = 3 mice/group). Green, α_v_ integrin; red, LAP; blue, activated fibroblasts (α-SMA); blue, epithelial cells (E-cadherin). Scale bar = 50 μm.

To validate the above findings *in vivo*, we examined production of LAP, α_v_ integrin, active TGF-β, and collagen in the lungs of WT and Del-1^−/−^ mice lungs following induction of PF. At 21 days post BLM administration (dpa), the lungs of Del-1^−/−^ mice showed greater colocalization of LAP and α_v_ integrins than those of WT mice; this colocalization was prevented by Del-1 supplementation (Figure 3A), indicating that Del-1 inhibits binding of α_v_ integrin to LAP. A prerequisite for this model is the proximity of α_v_ integrin, TGF-β, and Del-1 molecules. In the lungs of Del-1^−/−^ mice α_v_ integrin was localized in close proximity to LAP on both activated fibroblasts (labeled with an α-SMA antibody) and epithelial cells (labeled with an E-cadherin antibody) (Figure 3B).

### Del-1 Deficiency Activates TGF-β in Mice, Thereby Increasing Production of Collagen

Often, fibrosis develops independently of inflammation, but BLM induces inflammation-driven fibrosis in mice. To exclude involvement of inflammatory mediators prior to fibrosis, we employed a PF model in which TGF-β cDNA was directly introduced into mice via an adenovirus (20, 41).

**Figure 4:**
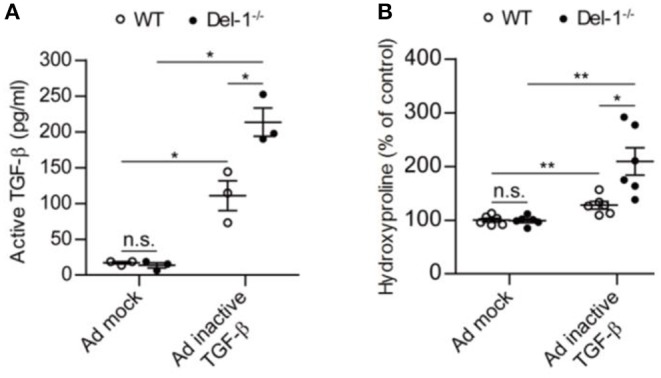
Endogenous Del-1 inhibits activation of TGF-β and subsequent collagen production. **(A)** Active TGF-β was measured in the BALF of WT and Del-1^−/−^ mice with PF induced by an adenovirus expressing inactive TGF-β. Mice received adenovirus intratracheally, and BALF was collected at 7 days post administration. Data are expressed as the mean ± SEM (*n* = 3 mice per group). *p < 0.05; n.s., not significant; Mann-Whitney *U*-test. **(B)** Hydroxyproline analysis of lung tissue from WT and Del-1^−/−^ mice with PF induced by adenovirus expressing inactive TGF-β. Lungs were collected at Day 14 post-adenovirus administration. The concentrations of hydroxyproline are shown as a percentage of the mean absolute concentration in WT mice treated with a mock adenovirus, which was set to 100%. Data are expressed as the mean ± SEM (*n* = 6 mice/group). *p < 0.05, **p < 0.01; n.s., not significant; Student's *t*-test.

At 7 days post intratracheal administration of an adenovirus expressing inactive TGF-β, WT and Del-1^−/−^ mice produced comparable levels of LAP (Figure S6). However, active TGF-β levels, and consequently, collagen production, were significantly higher in Del-1^−/−^ mice than in WT mice (Figure 4). By contrast, injection of active TGF-β-expressing adenovirus resulted in comparable levels of active TGF-β and collagen production in the BALF of WT and Del-1^−/−^ mice (Figure S7), indicating that Del-1 does not target active TGF-β directly, but rather targets a step during TGF-β activation.

Next, we tested whether Del-1 targets molecules downstream of TGF-β signaling *per se*. Del-1 treatment did not alter the expression of TGF-β receptors and Col1a by fibroblasts upon stimulation with TGF-β (Figures S8A,B). Levels of TGF-βR1 were comparable in resting lung fibroblasts from WT and Del-1^−/−^ mice (Figure S8C). Upon treatment of recombinant TGF-β (active TGF-β), the levels of phosphorylated TGF-βR1 and Smad2/3, which are markers of active TGF-β signaling, were not decreased in WT fibroblasts compared to those in Del-1^−/−^ fibroblasts (Figures S8D,E). Furthermore, TGF-β-induced proliferation and differentiation of fibroblasts into myofibroblasts was comparable in WT and Del-1^−/−^ mice (Figures S8F–H). Taken together, these findings indicate that Del-1 does not target downstream mediators of TGF-β signaling directly.

### Supplementation of Del-1 Attenuates Fibrosis in Mice

**Figure 5:**
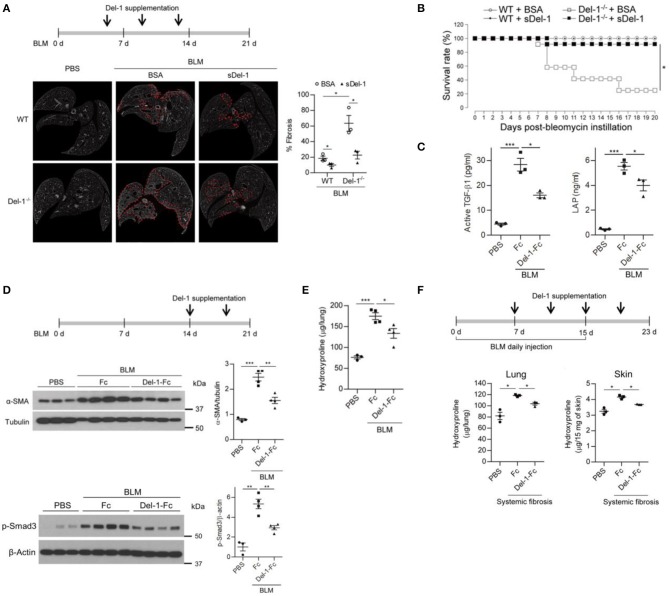
Supplementation with Del-1 attenuates the pathological characteristics of PF in mice. **(A)** Representative *ex vivo* micro-computed tomography (μ-CT) scans of lung sections from WT and Del-1^−/−^ mice with BLM (1 U/kg)-induced PF, which were supplemented intravenously with bovine serum albumin (BSA; 50 μg/dose) or soluble Del-1 (sDel-1; 50 μg/dose) at 5, 9, and 13 days post-BLM administration (dpa). Lung sections were taken at 21 dpa. Dashed lines indicate fibrotic regions. Quantification of fibrosis in the μ-CT images is shown in the right panel. Data are represented as the mean ± SEM (*n* = 3 mice per group). *p < 0.05; n.s., not significant; Mann-Whitney *U*-test. **(B)** Survival analysis of WT and Del-1^−/−^ mice with BLM-induced PF (1.5 U/kg BLM) that were supplemented with BSA or sDel-1 (intravenous injection, 50 μg/dose sDel-1 at 5, 9, and 13 dpa [*n* = 12 mice per group]). *p < 0.0001; log-rank test. **(C)** The levels of active TGF-β and LAP in the lungs of mice with BLM-induced PF that were supplemented with Fc or Del-1-Fc (intravenous injection; 50 μg/dose at 5, 9, and 13 dpa). The lung lysates were collected at 14 dpa and subject to ELISA. Data are expressed as mean ± SEM (*n* = 3 mice per group). *p < 0.05, ***p < 0.001; Mann-Whitney *U*-test. **(D)** Representative western blots to detect the expression of α-SMA and the level of phosphorylated Smad3 in WT mice with BLM-induced PF (2 U/kg BLM) supplemented with Fc or Del-1-Fc (intravenous injection; 50 μg/dose Fc or Del-1-Fc at 14, and 18 dpa). Lung lysates were collected at 21 dpa. Densitometric data are shown in the right panels. Data are expressed as mean ± SEM (*n* = 3–4 mice per group). **p < 0.01, ***p < 0.001; Student's *t*-test. **(E)** Hydroxyproline analysis of lung tissues from WT mice with BLM-induced PF (2 U/kg BLM) supplemented with Fc or Del-1-Fc, as in **(D)**. Data are expressed as the mean ± SEM (*n* = 3–4 mice per group). *p < 0.05, ***p < 0.001; Student's *t*-test. **(F)** Hydroxyproline analysis of lung and skin tissues from WT mice with systemic fibrosis (8 U/kg/dose BLM, daily subcutaneous injection for 15 days) supplemented with Fc or Del-1-Fc (intravenous injection; 50 μg/dose Del-1-Fc at 7, 11, 15, and 19 dpa after the first BLM instillation). Tissues were harvested at 23 days after the first BLM instillation. Data are expressed as the mean ± SEM (*n* = 3 mice per group). *p < 0.05; Mann-Whitney *U*-test.

In the BLM-induced PF model used herein, the level of TGF-β peaked during both the inflammatory and the fibrotic phases (Figure S5C). Of note, a second peak of active TGF-β production implies that a second or independent event that regulates TGF-β activation is necessary to control PF. Therefore, we asked whether Del-1 regulates fibrosis pathology during the course of BLM-induced PF. To evaluate whether Del-1 supplementation attenuates the fibrosis phenotype, we assessed pulmonary collagen deposition in BLM-induced PF mice supplemented with bovine serum albumin (BSA) or soluble Del-1 (sDel-1). As Del-1 limits leukocyte recruitment, and the inflammatory phase occurs earlier in the BLM-induced PF mouse model, we first gave mice sDel-1 to target the inflammatory phase. Supplementation with sDel-1 at 5, 9, and 13 dpa reduced pulmonary collagen deposition in both WT and Del-1^−/−^ mice with BLM-induced PF, as revealed by *ex vivo* micro-computed tomography (μ-CT) (Figure 5A) and a biochemical assay to detect expression of α-smooth muscle actin (SMA), an indicator of myofibroblasts (Figure S9A). Hypoxemia is a feature of end-stage PF (42). At 21 dpa, sDel-1 supplementation restored blood oxygen levels in WT mice with BLM-induced PF to levels similar to those in WT mice treated with PBS (Figure S9B). Furthermore, Del-1 supplementation led to a marked improvement in survival of Del-1^−/−^ mice with BLM-induced PF (Figure 5B). These data indicate that Del-1 limits inflammation during the early stage of BLM-induced PF development. Of note, Del-1 supplementation at 5, 9, and 13 dpa significantly decreased the levels of active TGF-β and LAP in the mice lung at 14 dpa (Figure 5C), demonstrating that Del-1 regulates TGF-β production *in vivo*. Given that Del-1 promotes the efferocytosis of apoptotic cells by macrophages and efficient apoptotic cell clearance contributes to the attenuation of fibrosis (43, 44), we looked into the levels of apoptotic neutrophils and epithelial cells in the tissue. The apoptosis of recruiting neutrophils was not significantly different between WT and Del-1^−/−^ mice at 7 dpa. Of note, the apoptosis of epithelial cells in the lung was increased in Del-1^−/−^ mice at 7 dpa (Figure S10A), However, Del-1 supplementation lowered the levels of the apoptosis of neutrophils and epithelial cells at 14 dpa in mice with BLM-induced PF (Figure S10B).

Next, we examined whether Del-1 regulates the early and late fibrotic phases of PF in addition to the inflammatory phase. To this end, sDel-1 was administered intravenously at 9 and 13 dpa (Figure S9C), or at 14 and 18 dpa (Figures 5D,E), and lung pathology was assessed. For both treatments, sDel-1 reduced α-SMA expression, collagen production, and the presence of fibrotic lung tissue significantly at 21 dpa (Figure S9C, Figures 5D,E). In addition, sDel-1 treatment reduced the phosphorylation of Smad3, indicating that TGF-β signaling is active in the tissues with BLM-induced PF, but that activation is reduced upon Del-1 supplementation (Figure 5D). Next, we examined whether Del-1 controls systemic fibrosis. To do this we used a model in which repetitive subcutaneous injections of high doses of BLM induced fibrosis in the lungs and skin (33). We found that, compared with control mice, collagen production in the lungs and skin of Del-1-treated mice fell significantly (Figure 5F). Collectively, these data demonstrate that sDel-1 ameliorates the pathologic characteristics of fibrosis.

### Plasma Del-1 Protein Levels Are Reduced in Patients With Idiopathic PF

**Figure 6:**
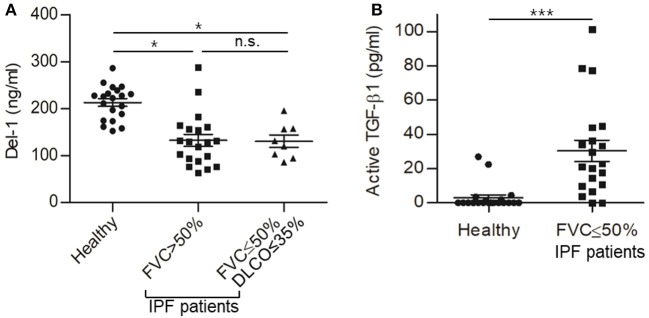
Reduced expression of Del-1 correlates with the development of PF in humans, which accompanies increased active TGF-β in plasma. **(A)** Del-1 protein levels in the plasma of healthy controls and PF patients: healthy controls (*n* = 20); mild-to-moderate PF (*n* = 20; FVC > 50%); and severe PF (*n* = 8; FVC ≤ 50% and DLCO ≤ 35%). Data are expressed as the mean ± SEM. *p < 0.05; n.s., not significant; Dunnett's test and Student's *t*-test. **(B)** Active TGF-β levels in the plasma of healthy controls (*n* = 20) and patients with PF (*n* = 20; FVC <50%). Data are expressed as mean ± SEM. ***p < 0.001; Student's *t*-test.

Finally, to examine the relevance of Del-1 to human fibrosis, we analyzed expression of Del-1 in plasma from patients with idiopathic PF (IPF). First, 40 patients diagnosed with IPF were classified into two groups according to their initial forced vital capacity (FVC) (FVC >50% and FVC ≤50%; *n* = 20 patients per group). In addition to FVC, we used another lung physiology variable, the diffusing capacity of the lung for carbon monoxide (DLCO), to classify the patients according to disease severity (45). Among the 20 patients with FVC ≤50%, patients with DLCO >35% were excluded. Thus, the mild-to-moderate IPF group comprised patients with FVC >50% and DLCO >35% (*n* = 20), while the severe IPF group comprised patients with FVC ≤50% and DLCO ≤35% (*n* = 8). In the mild-to-moderate group, FVC was 77.2% ± 3.2% and DLCO was 72.5% ± 6.3%; in the severe group, FVC was 39.5% ± 3.8% and DLCO was 29.0% ± 2.4%. Plasma levels of Del-1 in IPF patients were significantly lower than those in healthy controls (n = 20) (Figure 6A); however, there was no difference in levels between patients with mild-to-moderate IPF and those with severe IPF (Figure 6A). As expected, we found the inverse correlation of the levels of Del-1 with the levels of active TGF-β in the plasma of IPF patients (Figure 6B). These findings suggest an association between Del-1 and IPF, and indicate that reduced Del-1 levels may be an indicator of PF development. A summary of these results is presented in Figure S11.

## Discussion

Here, we used two mouse models of PF (BLM- and TGF-β-induced) to show that Del-1 inhibits activation of TGF-β by binding to α_v_β_6_ integrin. This study identifies a new function for Del-1, which can be added to the growing list. Studies show that Del-1 regulates osteoclastogenesis, myelopoiesis, and inflammation in an integrin-mediated manner (27, 43, 46).

Although our earlier work suggested a link between Del-1 and lung injury based on its inhibitory role on leukocyte migration (29), this study demonstrated that endogenous Del-1 inhibits latent TGF-β complexes by binding to integrin α_v_β_6_; thus, it prevents TGF-β activation, leading to fibrosis. That is, a new regulatory role for Del-1 in the development of this chronic disease model, independent of its anti-inflammatory role, is shown in this study. In addition, this study shows that Del-1 has a protective effect in multiple mouse models, including inflammation-independent fibrosis (adenoviral overexpression of inactive TGF-β in mice), as well as inflammation-dependent fibrosis (bleomycin-induced PF) and systemic fibrosis. More importantly, we show the association between Del-1/active TGF-β and idiopathic PF patients.

The mechanistic findings presented herein have important translational implications because Del-1 (the levels of which are reduced in human patients with PF) was able to reverse PF in an animal model. These findings are relevant in a clinical setting, making a strong case for further research into the therapeutic effect of Del-1 against PF.

Milk fat globule-EGF 8 (MFG-E8), which is structurally similar to Del-1, is a secreted glycoprotein comprising two N-terminal EGF-like domains and two C-terminal discoidin domains. MFG-E8 modulates inflammation and apoptotic cell clearance, thereby limiting tissue damage (47). Recent evidence suggests a role for MFG-E8 in PF (48). First, MFG-E8 levels are high in the lungs of patients with IPF. Second, lung injury increases MFG-E8 expression by alveolar macrophages, the alveolar interstitium, and the pulmonary endothelium. Third, the first discoidin domain of MFG-E8 binds to collagen, thereby facilitating collagen uptake. MFG-E8^−/−^ alveolar macrophages exhibit impaired uptake of Col1, leading to more severe PF, whereas recombinant MFG-E8 increases collagen uptake (48, 49). However, as shown herein, there was no clear difference between full-length Del-1 and Del-1 lacking the discoidin domains with respect to α_v_ integrin-mediated TGF-β activation or survival. This discrepancy requires further analysis. By contrast, another study reports that MFG-E8 levels are low in liver and serum from patients with cirrhosis, and that administration of mesenchymal stem cell-derived MFG-E8 alleviates liver fibrosis in mice by suppressing expression of TGF-βR1 by binding to α_v_β_3_ integrin (50). Although Del-1 binds to both α_v_β_6_ integrin and α_v_β_3_ integrin, we found that Del-1 supplementation had no effect on expression of TGF-β receptors, and that there were no differences in expression of TGF-β receptors between WT and Del-1^−/−^ fibroblasts. Additionally, Del-1 did not inhibit the downstream signaling of TGF-β in fibroblasts directly. These findings indicate that despite the structural similarity between Del-1 and MFG-E8, they play different roles; also, their expression levels and activities are regulated distinctly at the cellular and tissue levels under various pathophysiological conditions.

One of the tissue homeostatic roles of Del-1 is to resolve acute inflammation by promoting the efferocytosis of apoptotic neutrophils by macrophages. Del-1 (specifically, an RGD motif in the 2nd EGF domain in the N-terminus) binds to α_v_β_3_ on the surface of macrophages and it (specifically discoidin domains in the C-terminus) also binds to phosphatidylserine on apoptotic neutrophils, which bridges macrophages to neutrophils, thereby allowing macrophages to digest neutrophils (43). However, dysregulated efferocytosis is associated with chronic lung disease, such as fibrosis (44, 51). The apoptosis of recruiting neutrophils was not significantly different between WT and Del-1-deficient mice at 7 dpa. Of note, the apoptosis of epithelial cells in the lung increased in Del-1-deficient mice at 7 dpa, suggesting that endogenous Del-1 is not sufficient to regulate the apoptosis of neutrophils, whereas it affects the apoptosis of epithelial cells upon lung injury. However, Del-1 supplementation lowered the levels of apoptosis of neutrophils and epithelial cells in mice with BLM-induced PF. These data do not exclude a possibility that Del-1 at least partly regulates efferocytosis to attenuate PF, although it needs to be checked whether Del-1-mediated alveolar macrophages phagocytose apoptotic neutrophils or epithelial cells.

TGF-β binds to TGF-βR1 and the ligated TGF-βR1 activates a thread of Smad proteins (called the canonical signaling pathway) and Smad-independent pathways (called the non-canonical signaling pathways) to modulate cell functions. Both canonical and non-canonical TGF-β signaling pathways can induce fibrosis. Specifically, Smad proteins play distinct roles in the context of fibrosis; some Smads play profibrotic roles, whereas other Smads play antifibrotic roles. Smad3 is pro-fibrotic: phosphorylated Smad3 activates the transcription of ECM and α-SMA upon injury; Smad2 and Smad4 are regulators of Smad3-based gene transcription; and Smad7 is a negative feedback regulator of TGF-β/Smad canonical signaling: Smad7 competes with Samd2/3 for binding to activated TGF-βR1 and subsequent TGF-β/Smad signaling. Smad proteins interact with non-Smad co-activators and non-Smad co-suppressors. Furthermore, TGF-β/Smad signaling pathways interact with other pathways. In this regard, antifibrotic therapies targeting TGF-β include balancing between profibrotic and antifibrotic Smads and inhibiting non-canonical TGF-β signaling (52). On the other hand, TGF-β signaling may play anti-inflammatory roles in a context-dependent manner, by inducing regulatory T cells or inhibiting cytokine production and the proliferation and differentiation of immune cells (53).

TGF-β signaling is a crucial component to promote proliferation and differentiation of fibroblasts into myofibroblasts that produce ECM and limit inflammation in the course of fibrosis. In this regard, it is conceivable that maintaining the activity of TGF-β at a certain level in normal tissue might repress disease development. Addressing these aspects of TGF-β, therapeutic or preventive modalities will reduce fibrosis. In addition, disease period-specific application needs to be considered.

Given that TGF-β is a multi-functional cytokine involved in both physiology and immune defense, therapeutic approaches targeting TGF-β should be designed with caution (54, 55). In principle, indirect regulation of TGF-β activation or signaling, rather than direct blocking of TGF-β or its receptors, is desirable to fine-tune TGF-β functions. Approaches that target TGF-β, its receptor molecules, or its signaling pathways directly, or strategies that ablate TGF-β completely, have not been used for patients with dysregulated TGF-β signaling due to serious side effects such as cancer development. This is expected given that TGF-β signaling is critical for regulating immunity (6, 54). By contrast, Del-1 is less likely to cause serious side effects because Del-1-mediated inhibition of TGF-β signaling occurs only when the levels of both α_v_ integrin and inactive TGF-β are augmented (i.e., under pathologic conditions). This will greatly reduce inhibition of TGF-β signaling in non-target tissues.

Additionally, complete blockade of a specific integrin (as in β_6_-null mice), or inhibition of α_v_β_6_ integrin with high doses of a blocking antibody, aggravates lung inflammation (22, 56). Ablation of integrins or TGF-β may result in unexpected outcomes. Although blocking integrin-mediated TGF-β activation mimics the phenotype of TGF-β1-null mice, certain concentrations of α_v_-blocking monoclonal antibodies prevent PF (22). Thus, fine-tuning the conditions for TGF-β activation is essential. Del-1 regulates α_v_ integrin-mediated TGF-β activation but does not regulate the TGF-β molecule or its receptors directly. However, we found that although Del-1 bound specifically to LAP and α_v_ integrin, binding to LAP did not activate TGF-β directly in the absence of α_v_ integrin (Figures S1B, S4). Expression of α_v_ integrin is elevated during fibrosis, and Del-1 function depends on increased expression of α_v_ integrin. Therefore, Del-1 may control TGF-β activation under fibrosis-specific conditions. Furthermore, Del-1 was shown to regulate systemic fibrosis (Figure 5F). In conjunction with its known anti-inflammatory role in the lungs and other tissues, targeting Del-1 may also be a beneficial therapeutic approach for fibrotic diseases in other organs.

We observed lower levels of Del-1 protein in the plasma of PF patients than in that of healthy controls. However, plasma Del-1 did not decrease further as disease severity increased. In the mouse model, Del-1 levels decreased further in both the BALF and serum as PF progressed. In BLM-induced PF mice, Del-1 protein levels decreased slightly during the early phase (dominated by inflammation) and declined further during the fibrotic phase. The reason for the lack of a further decrease in PF patients as disease severity increased is unclear, but a couple of non-mutually exclusive possibilities may explain the discrepancy. First, initial FVC and DLCO values, according to which PF severity was determined, may not accurately reflect PF severity. Second, patients diagnosed with PF were beyond the early stage of fibrosis development; therefore, Del-1 levels may not change.

This study has several limitations. First, some *in vivo* data were obtained at a single time point, e.g., 21 dpa, at which peak fibrosis is observed in the BLM-induced fibrosis model (39, 40); therefore, it is not clear whether the effects observed are sustainable. Second, several experimental results are derived from a small number of mice because many of the mice died at the time of observation. Third, we did not examine the effect of Del-1 lacking the RGD motif. Nonetheless, we suspect that the RGD sequence might be involved in Del-1 function. Binding of Del-1 to α_v_ integrin results in TGF-β activation, whereas direct binding of Del-1 to LAP (whether the RGD sequence is involved or not) fails to activate TGF-β (in the absence of α_v_ integrin). Mapping a direct binding site for Del-1 on either α_v_β_6_ or to LAP should be the subject of a future study.

In conclusion, we propose a mechanism by which Del-1 protects against PF development. This occurs primarily through Del-1-mediated inhibition of α_v_β_6_ integrin-mediated TGF-β activation (independently of its inflammation-limiting mechanism), thereby exerting an anti-fibrotic effect in the lung. The results provide novel mechanistic insight into the role of Del-1 in fibrosis and lay a foundation for developing novel anti-fibrosis therapies.

## Data Availability Statement

All datasets generated for this study are included in the article/Supplementary Material.

## Ethics Statement

The animal studies were approved by the ASAN Institute for Life Sciences Institutional Animal Care and Use Committee (Project number: 2016-12-039). Human plasma from healthy control and PF patients was provided by the Collaborative Biobank of Korea at Soonchunhyang University Bucheon Hospital, Korea. All materials and clinical information used for research purposes (without personal identification) were collected from donors by the biobank; all donors provided written informed consent. Human studies were approved by the ASAN Medical Center Institutional Review Board (IRB no: 2017-1340) and Soonchunhyang University Bucheon Hospital Institutional Review Board (IRB no: schbc-biobank2017-013-01). The patients/participants provided their written informed consent to participate in this study.

## Author Contributions

D-YK and S-HL performed experiments, analyzed and interpreted data, and wrote the manuscript. YF, FJ, and J-AS performed experiments. HR, MK, and DL interpreted data. TL analyzed data. W-YK, S-BH, HC, M-SK, and C-OY provided important materials and helpful discussion. HH analyzed and interpreted data, and edited the manuscript. EC conceived the project, designed the research, interpreted data, supervised the research, and wrote the manuscript.

### Conflict of Interest

The authors declare that the research was conducted in the absence of any commercial or financial relationships that could be construed as a potential conflict of interest.
