# Supplementary material for: Del-1, an Endogenous Inhibitor of TGF-β Activation, Attenuates Fibrosis
Source: Front Immunol. 2020 Feb 7;11:68. doi: 10.3389/fimmu.2020.00068 (PMC7018852; doi:10.3389/fimmu.2020.00068)
Supplement: Supplementary file 1 [file Data_Sheet_1.docx]

**Title**

**Del-1, an endogenous inhibitor of TGF-β activation, attenuates fibrosis**

Del-1 is an anti-fibrotic factor

**Authors**

Dong-Young Kim^1,†^, Seung-Hwan Lee^1,†^, Yan Fu^1^, Feifeng Jing^1^, Won-Young Kim^2^, Sang-Bum Hong^3^, Jung-A Song^4^, Han Choe^4^, Hyun Jin Ryu^1^, Minjung Kim^1^, Dahae Lim^1^, Min-Seon Kim^5^, Chae-Ok Yun^6^, Taewon Lee^7^, Hoon Hyun^8^ and Eun Young Choi^1,*^

**Affiliations**

^1^Department of Biomedical Sciences, University of Ulsan College of Medicine, Asan Medical Center, Seoul, Republic of Korea.

^2^Division of Critical Care Medicine, Department of Internal Medicine, Chung-Ang University Hospital, Seoul, Republic of Korea.

^3^Division of Pulmonary and Critical Care Medicine, University of Ulsan College of Medicine, Asan Medical Center, Seoul, Republic of Korea.

^4^Department of Physiology, University of Ulsan College of Medicine, Seoul, Republic of Korea.

^5^Division of Endocrinology and Metabolism, Department of Internal Medicine, University of Ulsan College of Medicine, Asan Medical Center, Seoul, Republic of Korea.

^6^Department of Bioengineering, College of Engineering, Hanyang University, Seoul, Republic of Korea.

^7^Division of Applied Mathematical Sciences, College of Science and Technology, Korea University, Sejong, Republic of Korea.

^8^Department of Biomedical Sciences, Chonnam National University Medical School, Gwangju, Republic of Korea.

^†^These authors contributed equally to this work.

**^*^Corresponding author**

Eun Young Choi, Department of Biomedical Sciences, University of Ulsan College of Medicine, Asan Medical Center, 88 Olympic-Ro 43-Gil, Songpa-Gu, Seoul 05505, Korea.

Phone number: +82-2-3010-2208, E-mail: choieun@ulsan.ac.kr

**SUPPLEMENTARY MATERIAL**

FIGURE S1. Del-1 interferes with the binding of α_v_ integrins to LAP.

FIGURE S2. Representative qPCR (A) and flow cytometry (B) showing overexpression of αvβ_6_ integrin in HEK293T cells.

FIGURE S3. Production of LAP and active TGF-β by RAW 264.7 cells lentivirally expressing WT (inactive) or mutant (active) TGF-β.

FIGURE S4. Del-1 inhibits α_v_ integrin-mediated activation of TGF-β *in vitro*.

FIGURE S5. Production profiles of Del-1, collagen, TGF-β, α_v_ integrin α subunit, and inflammatory mediators in mice during the course of BLM-induced PF.

FIGURE S6. Levels of LAP in the BALF of WT and Del-1^-/-^ mice with PF induced by an adenovirus overexpressing inactive TGF-β.

FIGURE S7. Levels of active TGF-β and hydroxyproline in the lungs of mice with PF induced by an adenovirus overexpressing active TGF-β.

FIGURE S8. Expression of TGF-β receptors and activation of downstream targets of TGF-β signaling in primary fibroblasts.

FIGURE S9. Supplementation with Del-1 ameliorates the pathological characteristics of BLM-induced PF.

FIGURE S10. Del-1 regulates the apoptosis of epithelial cells and neutrophils in the lungs of mice with BLM-induced PF.

FIGURE S11. Schematic model of the role of Del-1 in PF.


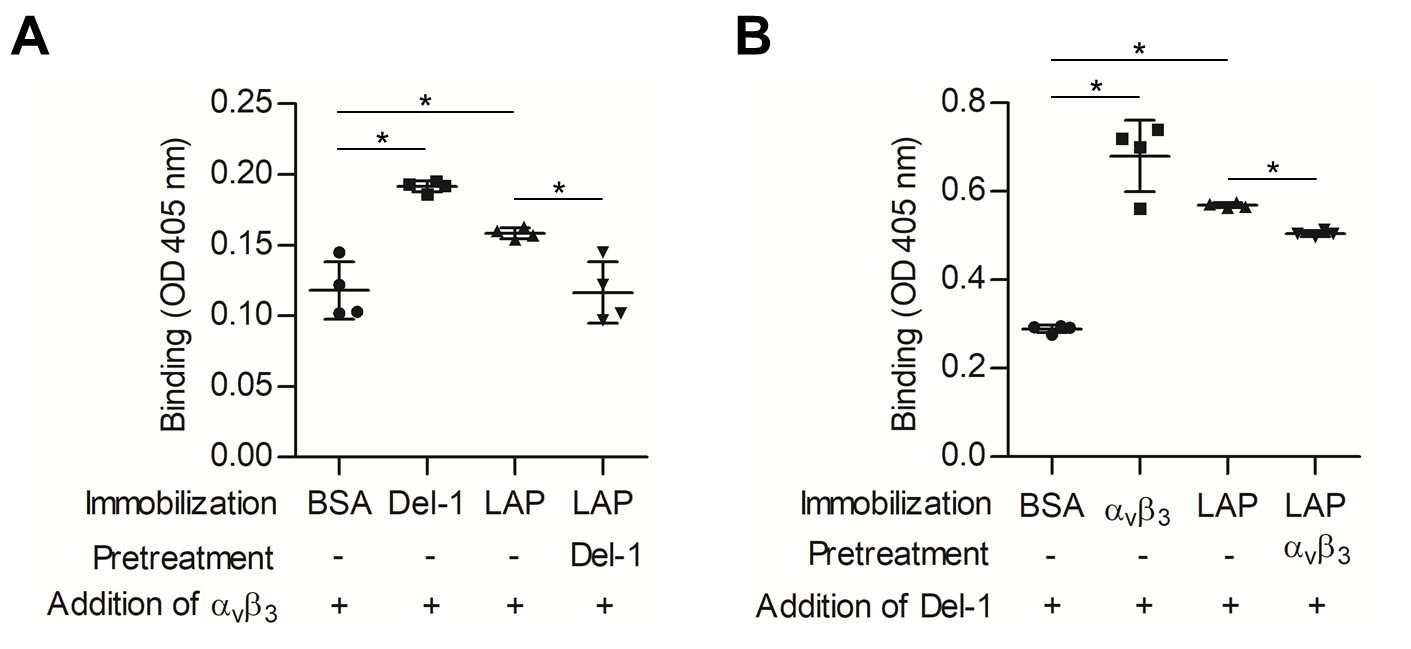


**FIGURE S1. Del-1 interferes with the binding of α_v_ integrins to LAP.**

**(A)** Binding of α_v_β_3_ integrin (100 nM) to immobilized BSA, Del-1 (100 nM), and LAP (100 nM), and binding of α_v_β_3_ integrin to immobilized LAP in the presence of Del-1, as assessed in a solid-phase binding assay. Data are representative of three independent experiments, each with similar results, and are expressed as the mean ± SD (n = 4 per group). **p* < 0.05; Student’s *t*-test. **(B)** Binding of Del-1 (100 nM) to immobilized BSA, α_v_β_3_ integrin (100 nM), and LAP (100 nM), and binding of Del-1 to immobilized LAP in the presence of α_v_β_3_ integrin, as assessed in a solid-phase binding assay. Data are representative of three independent experiments, each with similar results, and are expressed as the mean ± SD (n = 4 per group). **p* < 0.05; Student’s *t*-test.


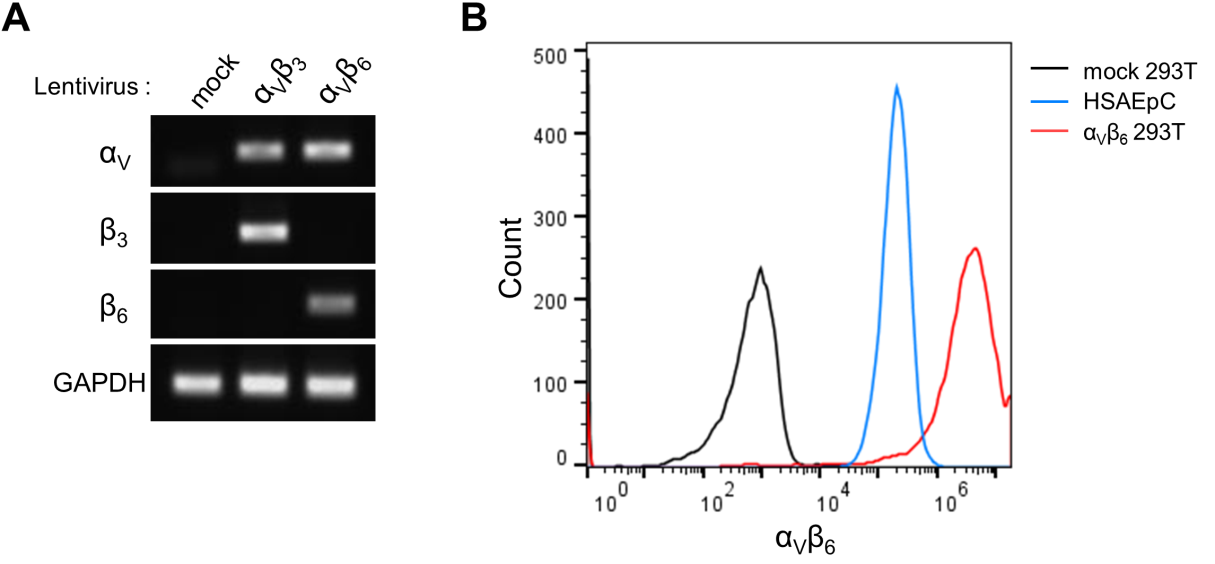


**FIGURE S2. Representative qPCR (A) and flow cytometry (B) showing overexpression of αvβ_6_ integrin in HEK293T cells.**


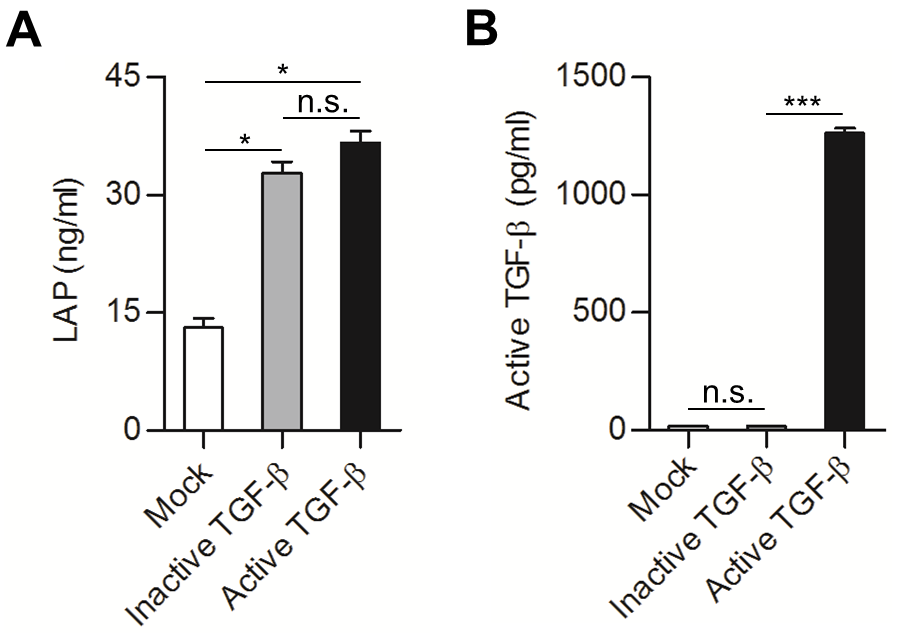


**FIGURE S3. Production of LAP and active TGF-β by RAW 264.7 cells lentivirally expressing WT (inactive) or mutant (active) TGF-β.**

**(A)** Levels of LAP and **(B)** active TGF-β in the culture supernatant of RAW 264.7 cells infected with a lentivirus expressing GFP (mock), inactive TGF-β, or active TGF-β. Infected RAW 264.7 cells (2 × 10^4^ cells/well) were plated in 96-well plates. After 24 h, the supernatants were analyzed by ELISA. Data are expressed as the mean ± SEM [*n* = 3 per group in **(A)**; *n* = 4 per group in **(B)**]. **p* < 0.05, ****p* < 0.001; n.s., not significant; Mann-Whitney U test **(A)** and Student’s *t*-test **(B)**.


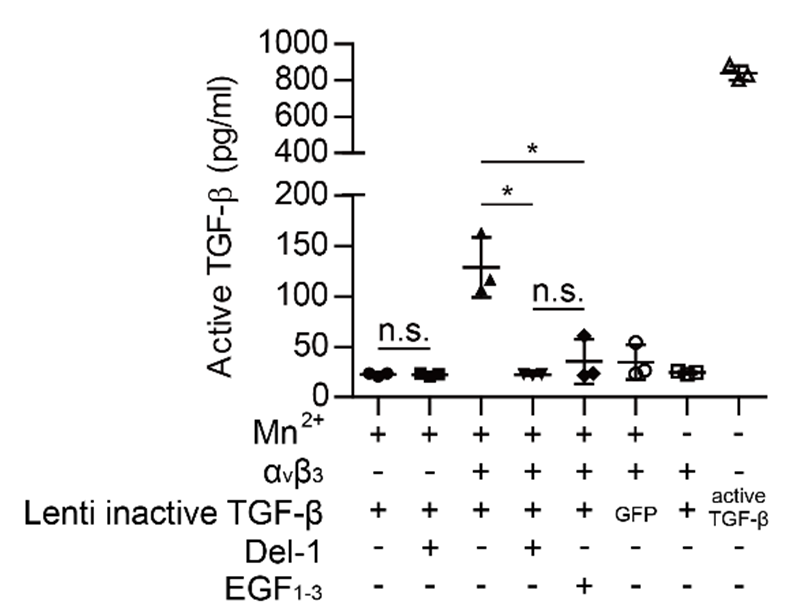


**FIGURE S4. Del-1 inhibits α_v_ integrin-mediated activation of TGF-β *in vitro*.**

Activation of TGF-β by immobilized α_v_β_3_ integrin in the absence or presence of Del-1. RAW264.7 cells overexpressing inactive TGF-β after lentiviral transduction were cultured for 16 h on plates coated with α_v_β_3_ integrin (5 μg/ml) for 3 h at 37 °C in a coating buffer containing 1 mM Mn^2+^. Del-1 was added 2 h before addition of 1 mM Mn^2+^. In some wells, a mutant version of Del-1 that contains three EGF domains but lacks the two discoidin domains (EGF_1−3_), was used instead of recombinant Del-1 (both at 5 μg/ml). Active TGF-β levels were measured in an ELISA 4 h after addition of Mn^2+^. Supernatant from RAW264.7 cells lentivirally overexpressing active TGF-β was used as a positive control. Data are representative of three independent experiments, each with similar results, and are expressed as the mean ± SD (n = 3 per group). **p* < 0.05; n.s., not significant; Mann-Whitney U test.


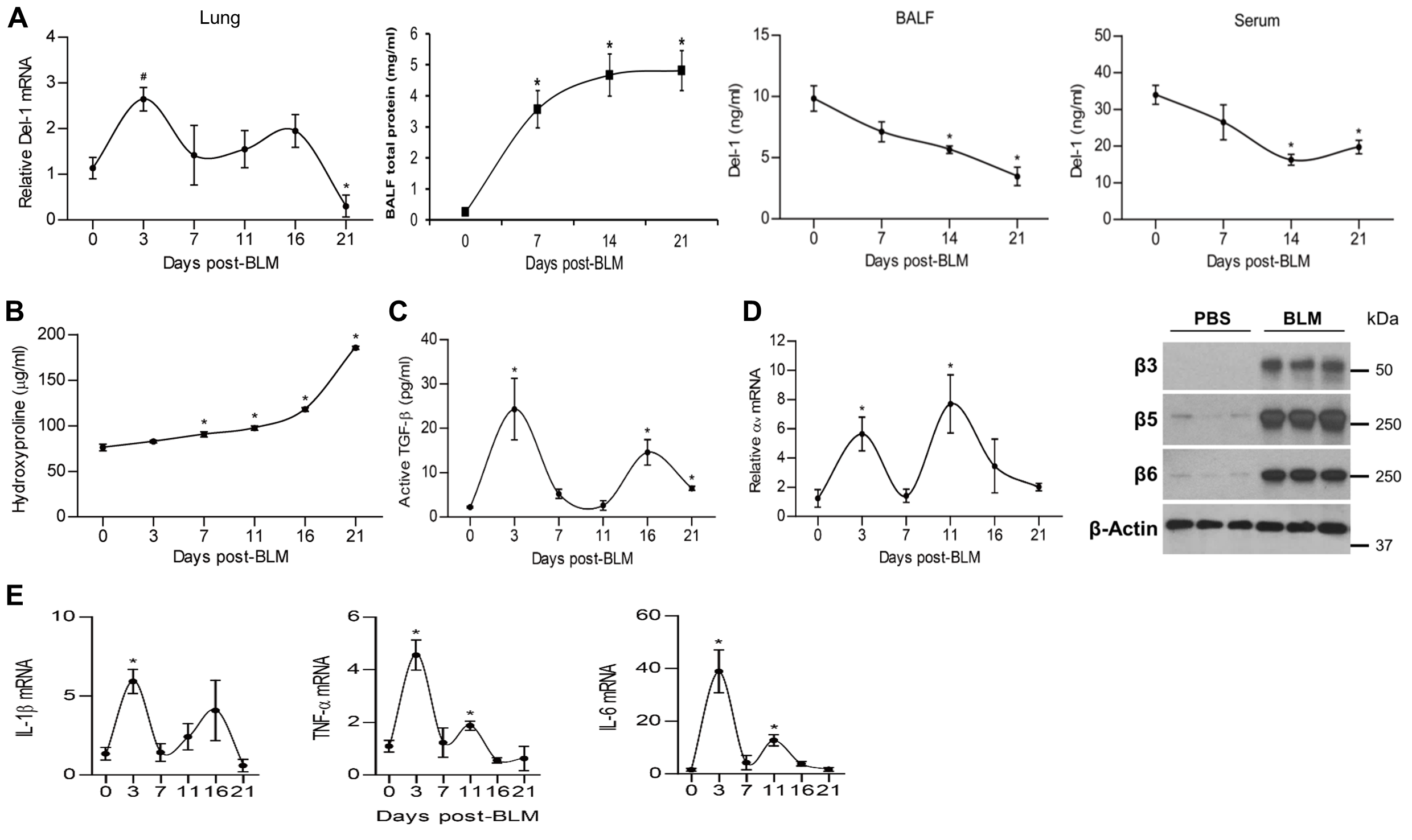


**FIGURE S5. Production profiles of Del-1, collagen, TGF-β, α_v_ integrin α subunit, and inflammatory mediators in mice during the course of BLM-induced PF.**

**(A)** Relative expression of Del-1 in lungs of WT mice during the course of BLM-induced PF (2 U/kg BLM). Total RNA was extracted from lung tissue at 0, 3, 7, 11, 16, and 21 dpa and mRNA encoding mouse Del-1 was detected by qRT-PCR. 18S mRNA was used for normalization. Expression of Del-1 at Day 0 was set to 1.0. Data are expressed as the mean ± SEM (n = 3–6 mice/group). **P* < 0.05; Student’s *t*-test. Levels of total protein present in bronchial alveolar lavage fluid (BALF) and concentrations of Del-1 protein in BALF and serum obtained at the indicated time points post-BLM (2 U/kg) administration in WT mice. Data are expressed as the mean ± SEM (Day 0, *n* = 5; Day 7, *n* = 5; Day 14, *n* = 4; and Day 21, *n* = 3). **p* < 0.05; Student’s *t*-test. **(B)** Hydroxyproline analysis of lung tissue from WT mice during the course of BiPF (2 U/kg BLM). Data are expressed as the mean ± SEM (Day 0, n = 3; Day 3, n = 3; Day 7, n = 3; Day 11, n = 4; Day 16, n = 4; Day 21, n = 2). **p* < 0.05; Student’s *t*-test or Mann-Whitney U test (for Day 0 *vs* Day 21, the sample size at 21 dpa was too small; however, individual values were markedly higher than those of the control group). **(C)** Levels of active TGF-β in the BALF of WT mice during the course of BLM-induced PF (2 U/kg BLM). Data are expressed as the mean ± SEM (n = 4 mice per group). **p* < 0.05; Student’s *t*-test. **(D)** Relative expression of α_v_ integrin in the lungs of WT mice during the course of BLM-induced PF (2 U/kg BLM), as analyzed by qRT-PCR. 18S mRNA was used for normalization. Expression of α_v_ integrin at Day 0 was set to 1.0. Data are expressed as the mean ± SEM (n = 3–6 mice/group). **P* < 0.05; Student’s *t* test. Representative western blots showing the beta subunits of a_v_ integrins in the lungs with BLM-induced PF. The samples were collected from three mice at 21 dpa.

**(E)** Production of proinflammatory cytokines during the course of BLM-induced PF in WT mice. Expression of mRNA encoding IL-1β, TNF-α, and IL-6 in the lungs of mice at the indicated time points post-BLM (2 U/kg) administration. Data are expressed as the mean ± SEM (n = 3–6 mice/group). **p* < 0.05; Student’s t-test.


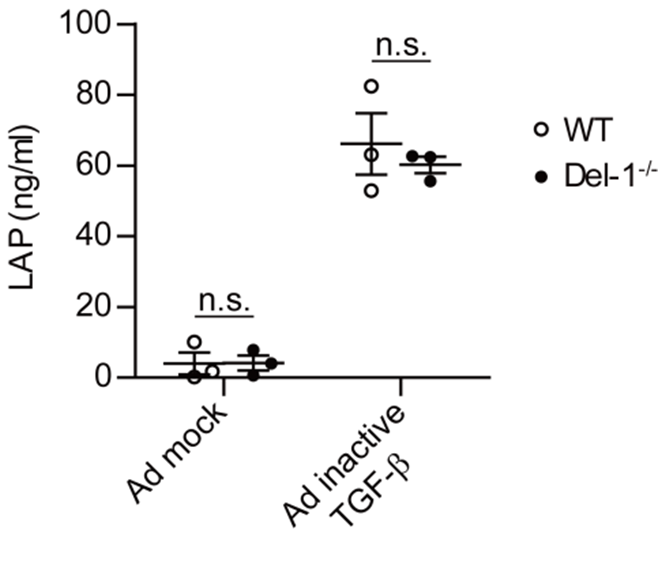


**FIGURE S6. Levels of LAP in the BALF of WT and Del-1^-/-^ mice with PF induced by an adenovirus overexpressing inactive TGF-β.**

Mice were administered adenovirus intratracheally. BALF was collected at 7 dpa and LAP concentrations were measured in an ELISA. Data are expressed as the mean ± SEM (*n* = 3 mice per group). n.s., not significant; Mann-Whitney U test.


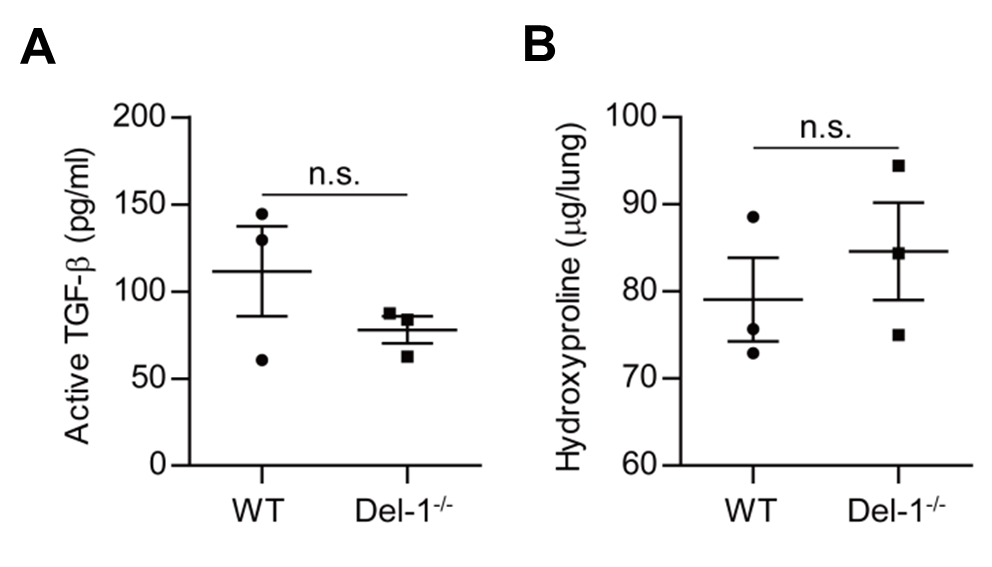


**FIGURE S7. Levels of active TGF-β and hydroxyproline in the lungs of mice with PF induced by an adenovirus overexpressing active TGF-β.**

**(A)** The levels of active TGF-β were measured in BALF from WT and Del-1^-/-^ mice with PF induced by active TGF-β-expressing adenovirus. Mice were administered adenovirus intratracheally. BALF was collected at Day 7 post-adenovirus administration. Data are expressed as the mean ± SEM (*n* = 3 mice per group). n.s., not significant; Mann-Whitney U test. **(B)** Hydroxyproline analysis of lung tissue from WT and Del-1^-/-^ mice with PF induced by adenovirus expressing active TGF-β. Lungs were collected at Day 14 post-adenovirus administration. Data are expressed as the mean ± SEM (*n* = 3 mice per group). n.s., not significant; Mann-Whitney U test.


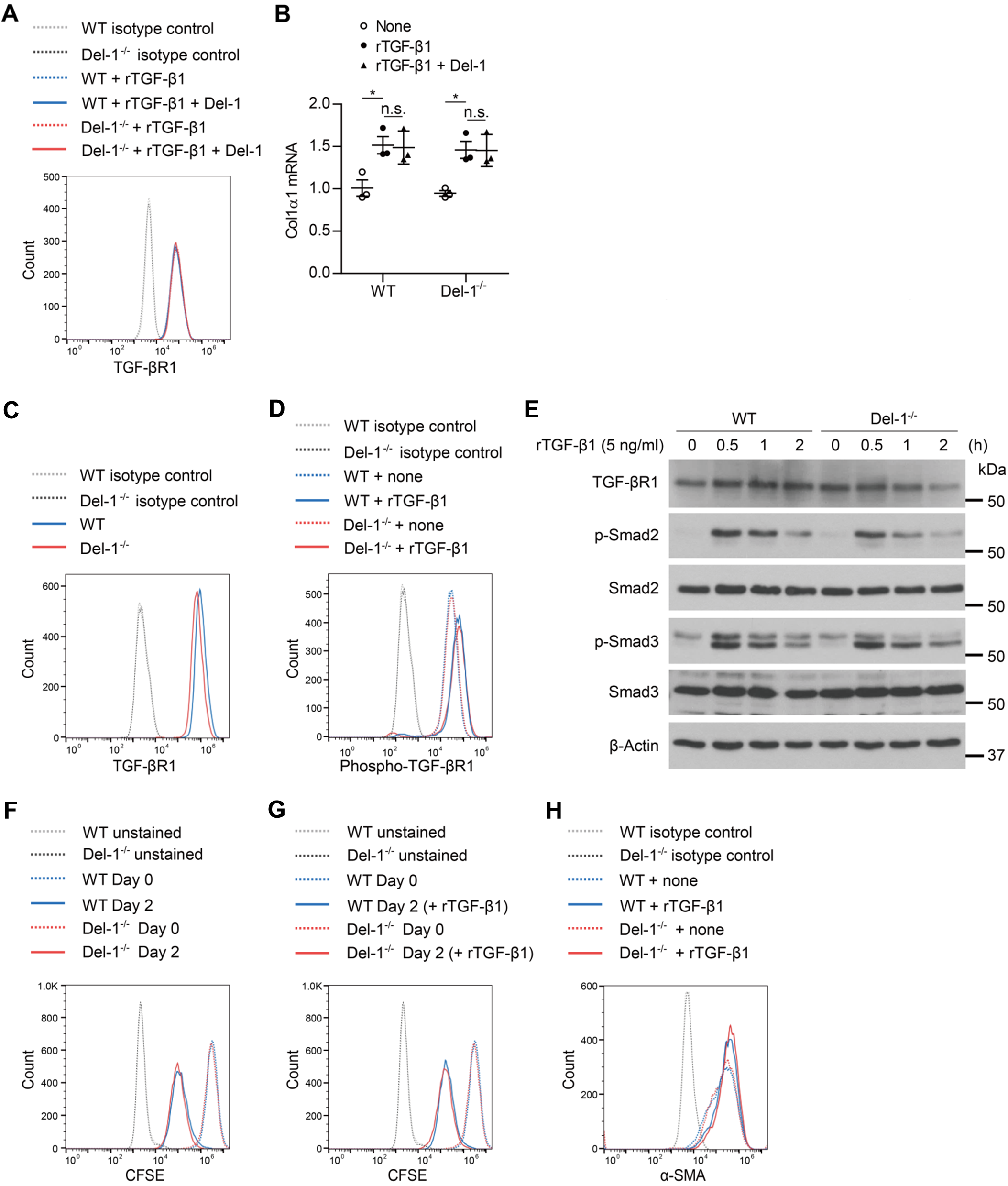


**FIGURE S8. Expression of TGF-β receptors and activation of downstream targets of TGF-β signaling in primary fibroblasts.**

**(A)** Representative histograms showing expression of TGF-βR1 upon Del-1 treatment by primary lung fibroblasts from WT and Del-1^-/-^ mice. Cells were incubated for 48 h in the presence of recombinant TGF-β (rTGF-β) (5 ng/ml) and Del-1 (0.5 μg/ml), stained, and analyzed by flow cytometry. Data shown are representative of three independent experiments. **(B)** Relative expression of *col1a* by primary lung fibroblasts treated with Del-1, as determined by qRT-PCR. Cells were incubated for 24 h in the presence of rTGF-β (5 ng/ml) and Del-1 (0.5 μg/ml). 18S mRNA was used for normalization. Gene expression in non-treated WT fibroblasts was set to 1.0. Data are expressed as the mean ± SD (n = 3 per group). *, *p* < 0.05; n.s., not significant; Mann-Whitney U test. **(C,D)** Representative histograms showing expression of TGF-βR1 **(C)** and phospho-TGF-βR1 **(D)** by primary lung fibroblasts from WT and Del-1^-/-^ mice. Cells were incubated for 30 min in the absence or presence of rTGF-β (5 ng/ml), and then stained and analyzed by flow cytometry. Data shown are representative of three independent experiments. **(E)** Expression of TGF-βR1 and phospho-Smad2/3 protein expression by primary lung fibroblasts from normal WT and Del-1^-/-^ mice. Cells were treated with rTGF-β (5 ng/ml) for the indicated times, and lysates were analyzed by western blotting. The blot shown is representative of three independent experiments. **(F,G)** Representative histograms showing proliferation **(F)** and differentiation **(G)** of primary lung fibroblasts from WT and Del-1^-/-^ mice. Cells were stained with carboxyfluorescein diacetate succinimidyl ester (CFSE) and incubated for 48 h in the absence **(F)** or presence **(G)** of rTGF-β (5 ng/ml). CFSE intensity was analyzed by flow cytometry. Data shown are representative of three independent experiments. **(H)** Representative histograms showing differentiation of primary fibroblasts from WT and Del-1^-/-^ mice. Cells were plated overnight, incubated for 24 h with 2.5% serum-containing medium, and then treated for 24 h with rTGF-β (5 ng/ml). The cells were then stained to examine α-SMA expression by flow cytometry. Data are representative of three independent experiments.


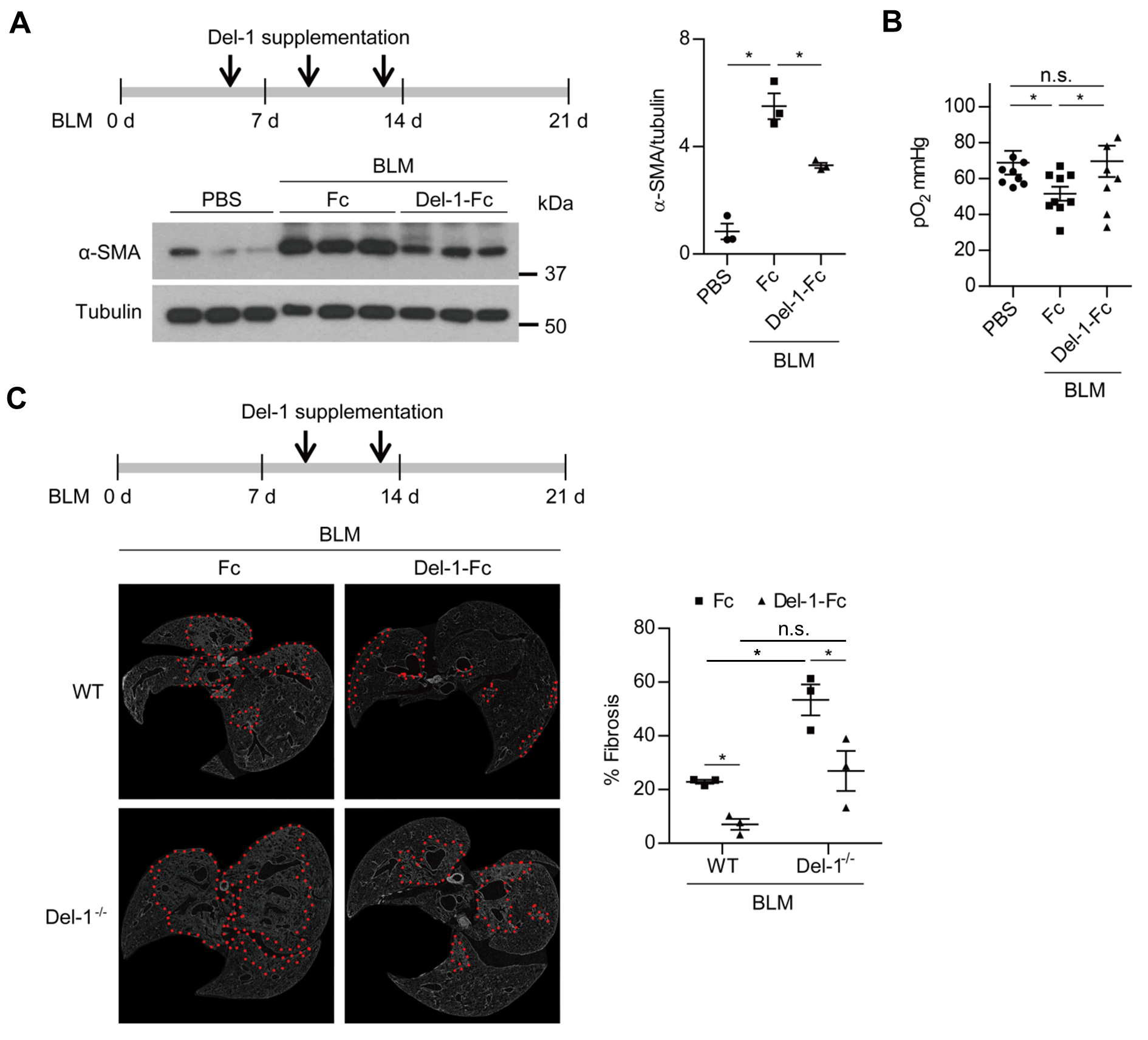


**FIGURE S9. Supplementation with Del-1 ameliorates the pathological characteristics of BLM-induced PF.**

**(A)** Representative western blot showing expression of α-SMA by WT and Del-1^-/-^ mice with BLM-induced PF (2 U/kg BLM) supplemented with Fc or Del-1-Fc (intravenous injection, 50 μg/dose Del-1-Fc at 5, 9, and 13 dpa). Lung lysates were collected at 21 dpa. Densitometric data are shown in the right panel. Data are expressed as the mean ± SEM (*n* = 3 mice per group). **p* < 0.05; Mann-Whitney U test. **(B)** Blood oxygen levels of WT mice with BLM-induced PF (2 U/kg BLM) supplemented with Fc or Del-1-Fc (intravenous injection, 50 μg/dose Del-1-Fc at 5, 9, and 13 dpa). Blood was collected at 21 dpa. Data are expressed as the mean ± SEM (*n* = 9–10 mice per group). **p* < 0.05; n.s., not significant; Student’s *t*-test. **(C)** Representative *ex vivo* micro-computed tomography (μ-CT) images of lung sections from WT and Del-1^-/-^ mice with BLM-induced PF that were supplemented with Fc or Del-1-Fc (intravenous injection; 50 μg/dose Fc or Del-1-Fc at 9 and 13 dpa). Lung sections were collected at 21 dpa. Dashed lines indicate fibrotic areas. Quantification of fibrosis in the μ-CT images is shown in the right panel. Data are expressed as the mean ± SEM (*n* = 3 mice per group). **p* < 0.05; Mann-Whitney U test.


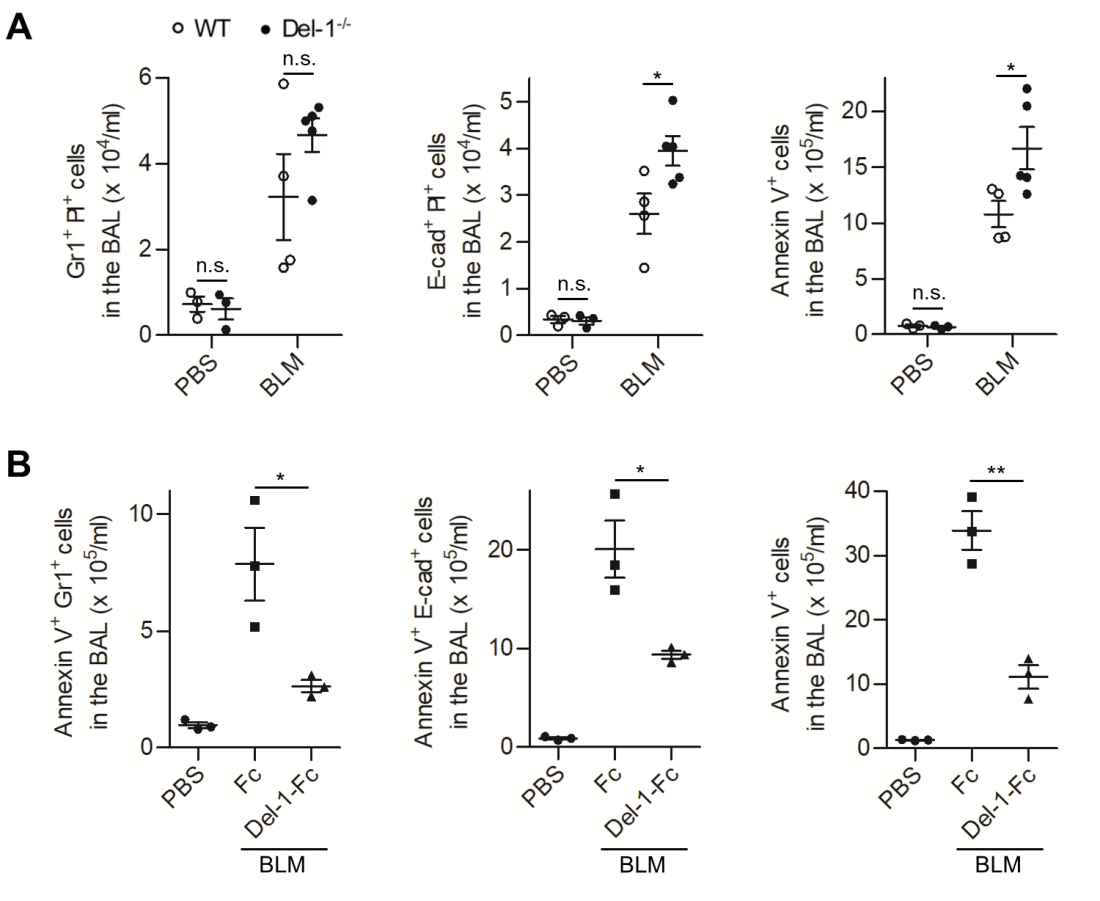


**FIGURE S10. Del-1 regulates the apoptosis of epithelial cells and neutrophils in the lungs of mice with BLM-induced PF.**

**(A)** Number of apoptotic epithelial cells (E-cad^+^) and neutrophils (Gr1^+^) in the BALF of mice with BLM-induced PF at 7 dpa. **(B)** Number of apoptotic epithelial cells and neutrophils in the BALF of mice with BLM-induced PF at 14 dpa, the mice being supplemented with Fc or Del-1-Fc (intravenous injection, 50 μg/dose at 5, 9, and 13 dpa). Data are expressed as mean ± SEM (n = 3–5 mice per group). *p < 0.05, **p < 0.01, n.s., not significant; Mann-Whitney U test.


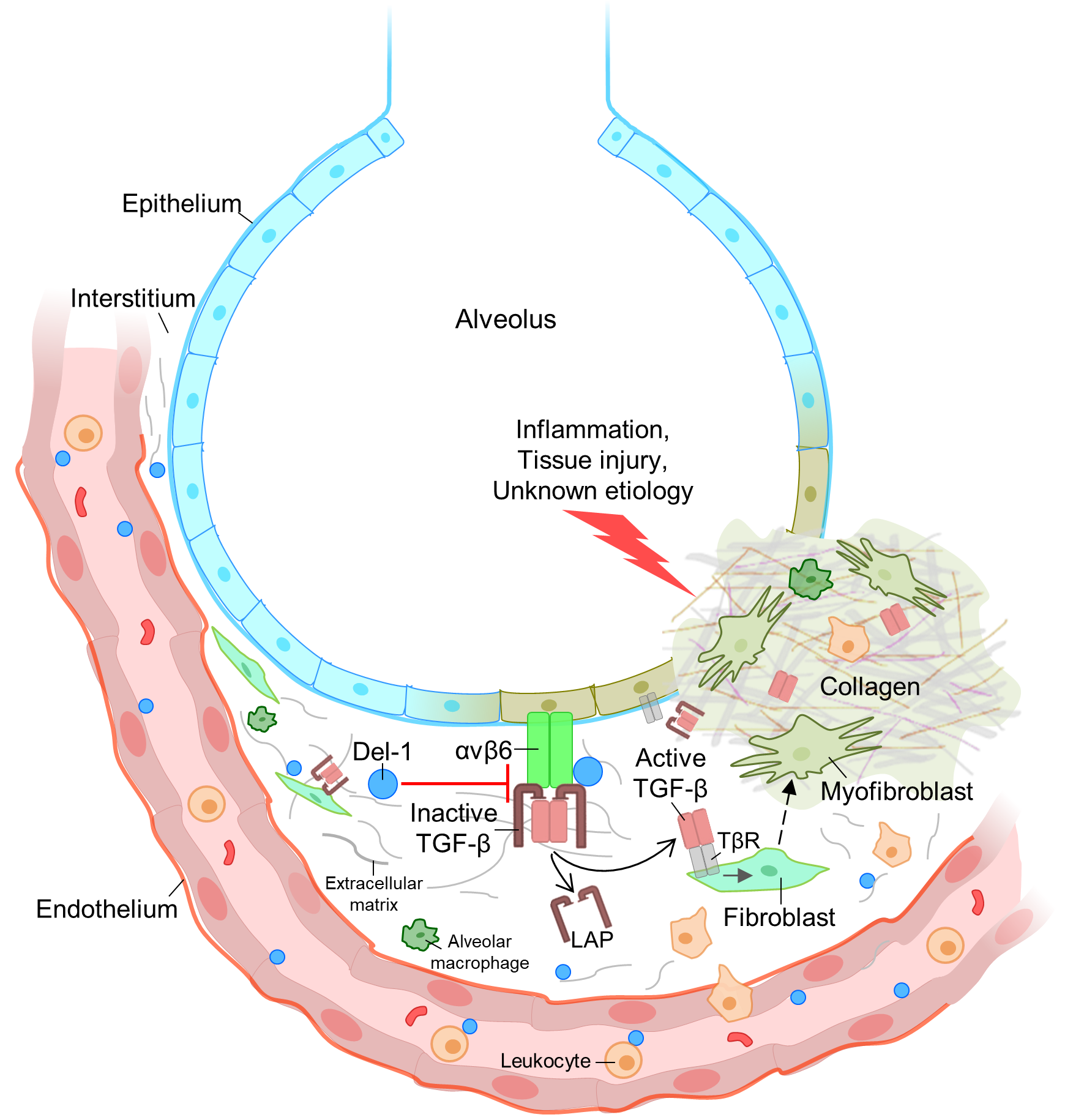


**FIGURE S11. Schematic model of the role of Del-1 in PF.**
